# Solution structure of extracellular loop of human β4 subunit of BK channel and its biological implication on ChTX sensitivity

**DOI:** 10.1038/s41598-018-23016-y

**Published:** 2018-03-15

**Authors:** Yanting Wang, Wenxian Lan, Zhenzhen Yan, Jing Gao, Xinlian Liu, Sheng Wang, Xiying Guo, Chunxi Wang, Hu Zhou, Jiuping Ding, Chunyang Cao

**Affiliations:** 1 State Key Laboratory of Bioorganic and Natural Product Chemistry, Center for Excellence in Molecular Synthesis, Shanghai Institute of Organic Chemistry, Chinese Academy of Sciences (CAS), 345 Lingling Road, Shanghai, 200032 China; 2 Key Laboratory of Molecular Biophysics of the Ministry of Education, College of Life Science and Technology, Huazhong University of Science and Technology, 1037 Luoyu Road, Wuhan, 430074 Hubei China; 3 Department of Analytical Chemistry, Shanghai Institute of Materia Medica, CAS, 555 Zuchongzhi Road, Shanghai, 201203 China; 4 University of Chinese Academy of Sciences, No. 19(A) Yuquan Road, Shijingshan District Beijing, 100049 China; 5 Collaborative Innovation Center of Chemistry for Life Sciences, Chinese Academy of Sciences (CAS), 345 Lingling Road, Shanghai, 200032 China

## Abstract

Large-conductance Ca^2+^- and voltage-dependent K^+^ (BK) channels display diverse biological functions while their pore-forming α subunit is coded by a single *Slo1* gene. The variety of BK channels is correlated with the effects of BKα coexpression with auxiliary β (β1-β4) subunits, as well as newly defined γ subunits. Charybdotoxin (ChTX) blocks BK channel through physically occluding the K^+^-conduction pore. Human brain enriched β4 subunit (hβ4) alters the conductance-voltage curve, slows activation and deactivation time courses of BK channels. Its extracellular loop (hβ4-loop) specifically impedes ChTX to bind BK channel pore. However, the structure of β4 subunit’s extracellular loop and the molecular mechanism for gating kinetics, toxin sensitivity of BK channels regulated by β4 are still unclear. To address them, here, we first identified four disulfide bonds in hβ4-loop by mass spectroscopy and NMR techniques. Then we determined its three-dimensional solution structure, performed NMR titration and electrophysiological analysis, and found that residue Asn123 of β4 subunit regulated the gating and pharmacological characteristics of BK channel. Finally, by constructing structure models of BKα/β4 and thermodynamic double-mutant cycle analysis, we proposed that BKα subunit might interact with β4 subunit through the conserved residue Glu264(BKα) coupling with residue Asn123(β4).

## Introduction

**Figure 1:**
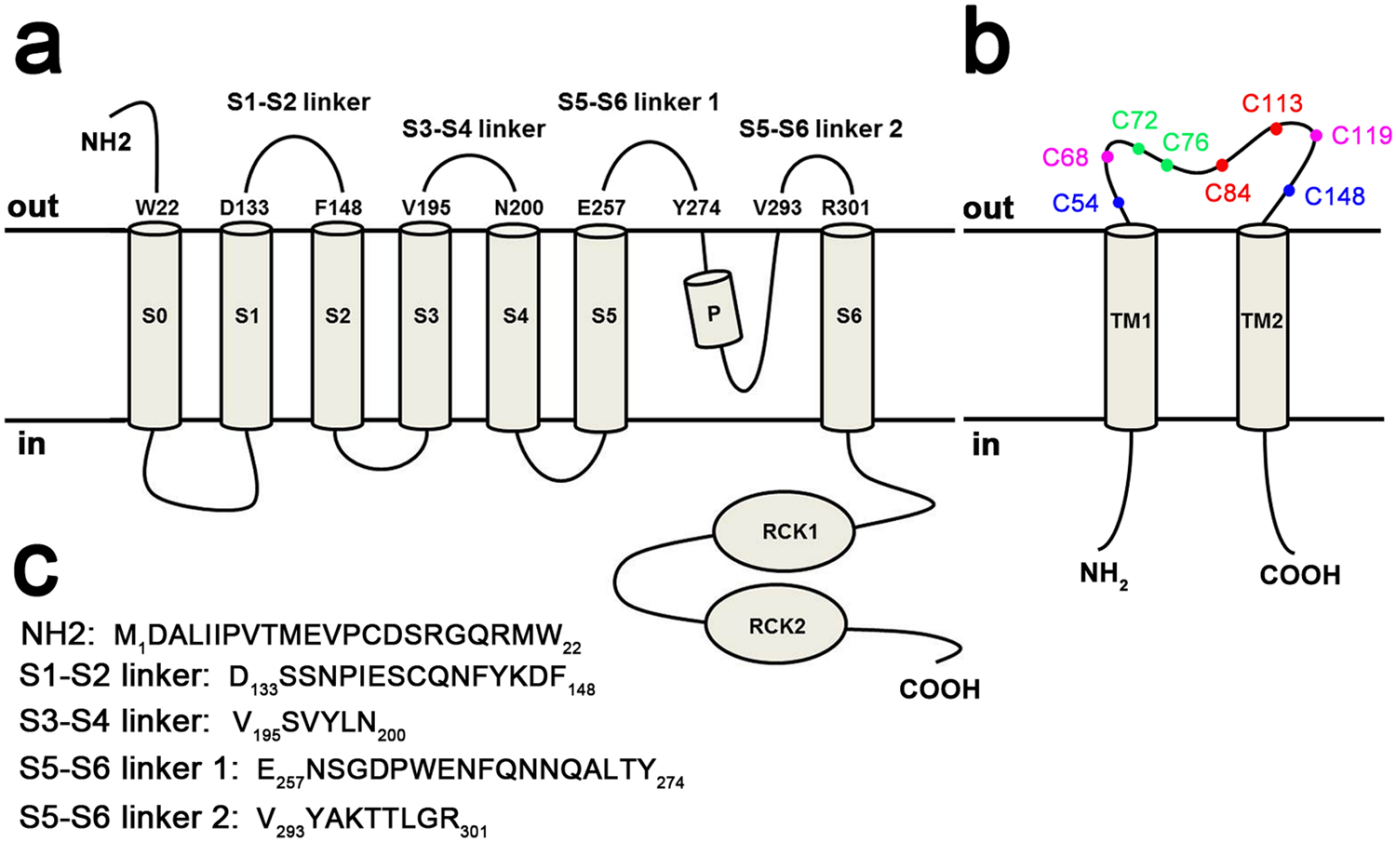
The topology of (**a**) BKα subunit and (**b**) β4 subunit. The residue numbers of (**c**) five extracellular segments (used in NMR titration experiments) in BKα subunit were highlighted, and the amino acid sequences of these segments were listed in (**c**). In (**b**), the eight cysteines were also marked in the extracellular loop of β4 subunit.

Large-conductance Ca^2+^- and voltage-dependent K^+^ (BK) channels display diverse biological functions in different cells and tissues. The functional diversity arises mainly from the channel pore-forming α subunits assembling with up to four tissue-enriched auxiliary β subunits (β1-β4), as well as newly defined γ subunits^1–3^. As shown in Fig. 1a, BKα subunit is encoded by a single gene (*Slo1*)^4^, containing seven transmembrane helices (S0-S6) and two C-terminal RCK domains^4,5^, while regulatory β subunits share a putative membrane topology with two transmembrane segments (TM) connected by a sizeable, extracellular loop and with N- and C-termini oriented toward the cytoplasm. The β subunits are responsible for a variety of the kinetic and pharmacological characteristics of BK channels in native tissues. β1 subunit increases the apparent Ca^2+^/voltage sensitivity of BK channel, and affects the contractility of the vascular smooth muscle cells. β2 and β3b subunits not only modulate the activation of BK channels, but also produce faster inactivating currents^6^. Human β4 subunit (hβ4) is enriched in brain^1,2,7–9^, which slows both activation and deactivation kinetics of BK channels^2,7^, and causes negative shifts of the channels’ conductance-voltage (G-V) curves at high Ca^2+^ concentration (>10 μM), but it leads to positive shifts at a low Ca^2+^ concentration^10–12^, suggesting that it plays a critical role in regulating neuronal excitability and neurotransmitter release^13^. Typically, four Slo1 turrets decentralize distally from BK channel pore to provide a wide open conformer, and charybdotoxin (ChTX) blocks the Slo1 alone channel by occluding the K^+^-conduction pore^14,15^. However, it’s reported that the extracellular loop of β4 (β4-loop) prevents ChTX from binding Slo1/β4 channels^1,16–19^. The physical association between BKα and β4 subunits or the molecular mechanism for pharmacological and gating regulation of BK channel by β4 remains unclear. In addition, the extracellular loop of human β4 subunit (hβ4-loop) contains eight cysteines (Fig. 1b), which were suggested to form four S-S bonds, and easily result in protein aggregation. Therefore, how these cysteines form disulfide bonds to stabilize the conformation is also kept unclear.

To address these questions, in this report, we first identified four S-S bonds in hβ4-loop by mass spectroscopy and NMR techniques, and determined the three-dimensional (3D) NMR structure of free hβ4-loop. Then through NMR titration experiments and electrophysiological studies, we found that residue Asn123, close to the three reported basic residues Lys120, Arg121 and Lys125^20^, locating in a flexible loop of hβ4-loop structure, regulated the channel gating kinetics in typical Ca^2+^ concentration of 10 μM, as well as the ChTX sensitivity of BK channel. Finally, based on the molecular modeling and double mutant cycle analysis, we inferred that residue Asn123 of β4 subunit might interact with the conserved residue Glu264 of BKα subunit. These results shed light on the unique properties of BK channel imparted by β4 subunit and may accelerate the process of designing specific drugs in the future.

## Results

### Determination of disulfide bonds and solution structure of hβ4-loop

**Figure 2:**
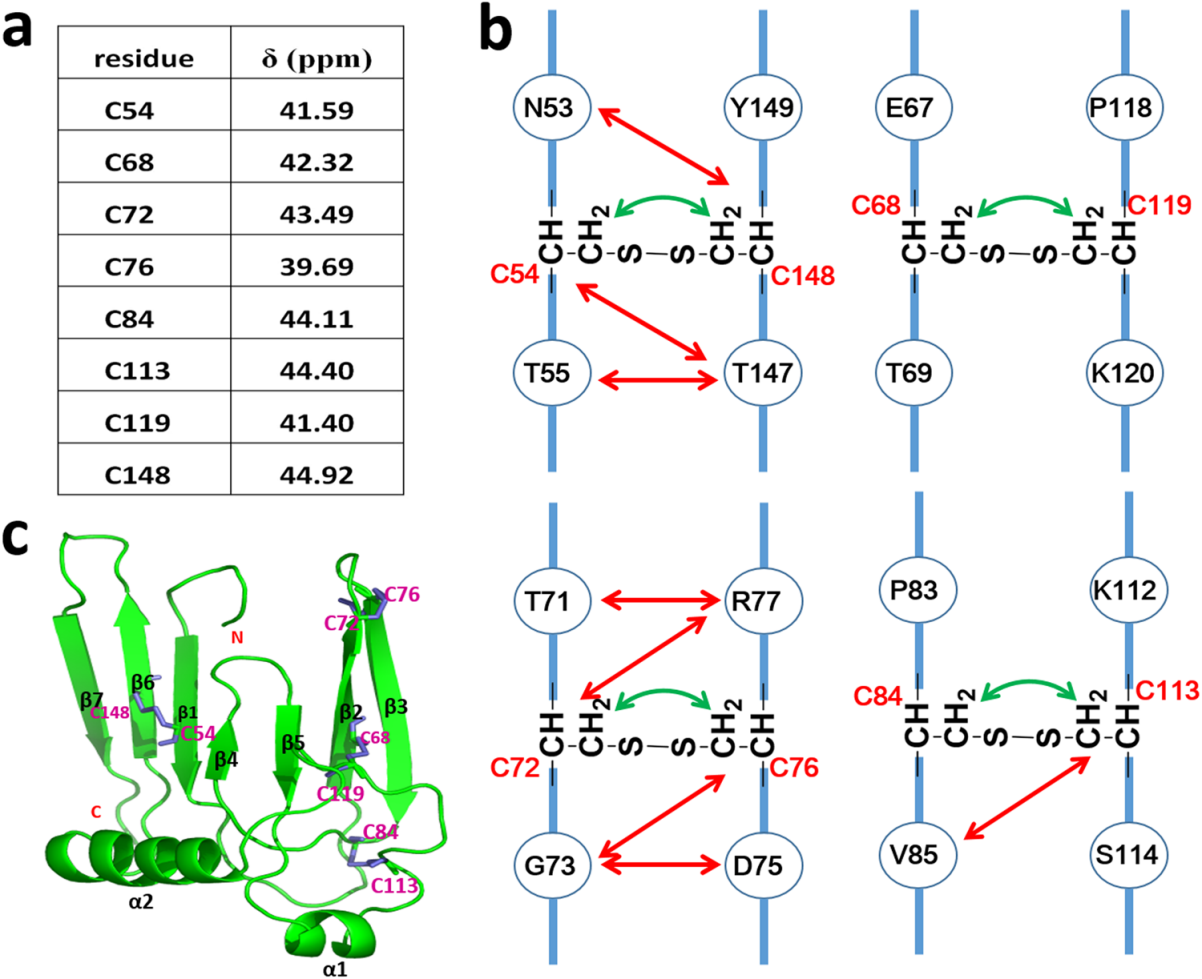
The S-S bond analyzed by NMR techniques. (**a**) NMR chemical shifts of Cβ atoms of all cysteines in hβ4-loop indicate all these cysteines are involved to form S-S bonds. (**b**) The NOEs observed between protons in the cysteines (displayed by green arrows) or the residues adjacent to cysteines (displayed by red arrows), supporting all S-S bonds formation. (**c**) Ribbon representation of the structure. The S-S bonds and the 2^nd^ structural elements were highlighted in pink and black, respectively.

The eight cysteines of hβ4-loop were known to form four disulfide bonds, which govern the topology of hβ4-loop, stabilize its conformation, and affect its biological functions^7^. Therefore, it’s necessary to determine how these cysteines form S-S bonds. Generally, the NMR signal of the Cβ atom of the reduced cysteine (*i.e*., free cysteine state) is always resonated at a higher field (close to 30 ppm), while the NMR signal of the Cβ atom of the oxidized cysteine (*i.e*., S-S state) is resonated at a lower field (near or larger than 40 ppm). Thus, NMR is a common technique to probe whether cysteines form disulfide bond. Through a series of three-dimensional (3D) experiments, including HNCA, HNCO, HNCACB and CBCACONH, we successfully assigned about 96% of the resonances of the backbone atoms of hβ4-loop. As shown in Fig. 2a, the chemical shifts of the Cβ atoms of residues Cys54, Cys68, Cys72, Cys76, Cys84, Cys113, Cys119 and Cys148 in hβ4-loop were assigned at 41.59 ppm, 42.32 ppm, 43.49 ppm, 39.69 ppm, 44.11 ppm, 44.40 ppm, 41.38 ppm and 44.92 ppm, respectively, indicating that all these cysteines form disulfide bonds.

To characterize how these cysteines form S-S bonds, high-throughput mass spectrometry, termed as pLink-SS^21^, was firstly performed. Two disulfide bonds between Cys68 and Cys119 and between Cys54 and Cys148 were clearly mapped, as shown in Figure S1. The left two disulfide bonds formed among Cys68, Cys72, Cys76 and Cys119 were then determined based on the results of NOE assignments and secondary structure prediction, which will be described below. According to the backbone atoms assignments, the fragments in the regions of Thr50-Val56, Phe66-Gly73, Cys76-Tyr82, Val89-Asn91, Asn95-Leu99, Phe146-Asn151 and Asp158-Arg163 were predicted to fold into seven β-sheets by TALOS program^22,23^. Through a series of 3D experiments for side-chain atoms’ resonances and NOEs assignment, such as HCCH-TOCSY, ^15^N-edited HSQC-TOCSY, ^15^N or ^13^C-edited HSQC-NOESY, about 70% side-chain atoms resonances of hβ4-loop were obtained. The observed NOE’s pattern between two fragments (*i.e*, Phe66-Gly73 and Cys76-Tyr82) suggested that they fold into anti-parallel sheets (Figure S2), which was further confirmed by 3D structure of hβ4-loop discussed below. In space, Cys72 is near to Cys76, suggesting that they pair together. Therefore, residues Cys84 and Cys113 form another disulfide bond, which was confirmed by NOEs observed between Hβ atoms of Cys84 and of Cys113, and between Val85 methyl groups and Cys113 Hβ atoms (Fig. 2b). During NOE assignments, we also observed NOEs between Hβ atoms of Cys72 and Cys76, between Hβ atoms of Cys68 and Cys119, and between Hβ atoms of Cys54 and Cys148 (Fig. 2b,c), which further characterized S-S bond formation between these cysteines, consistent with the results from pLink-SS technique mentioned above.

**Figure 3:**
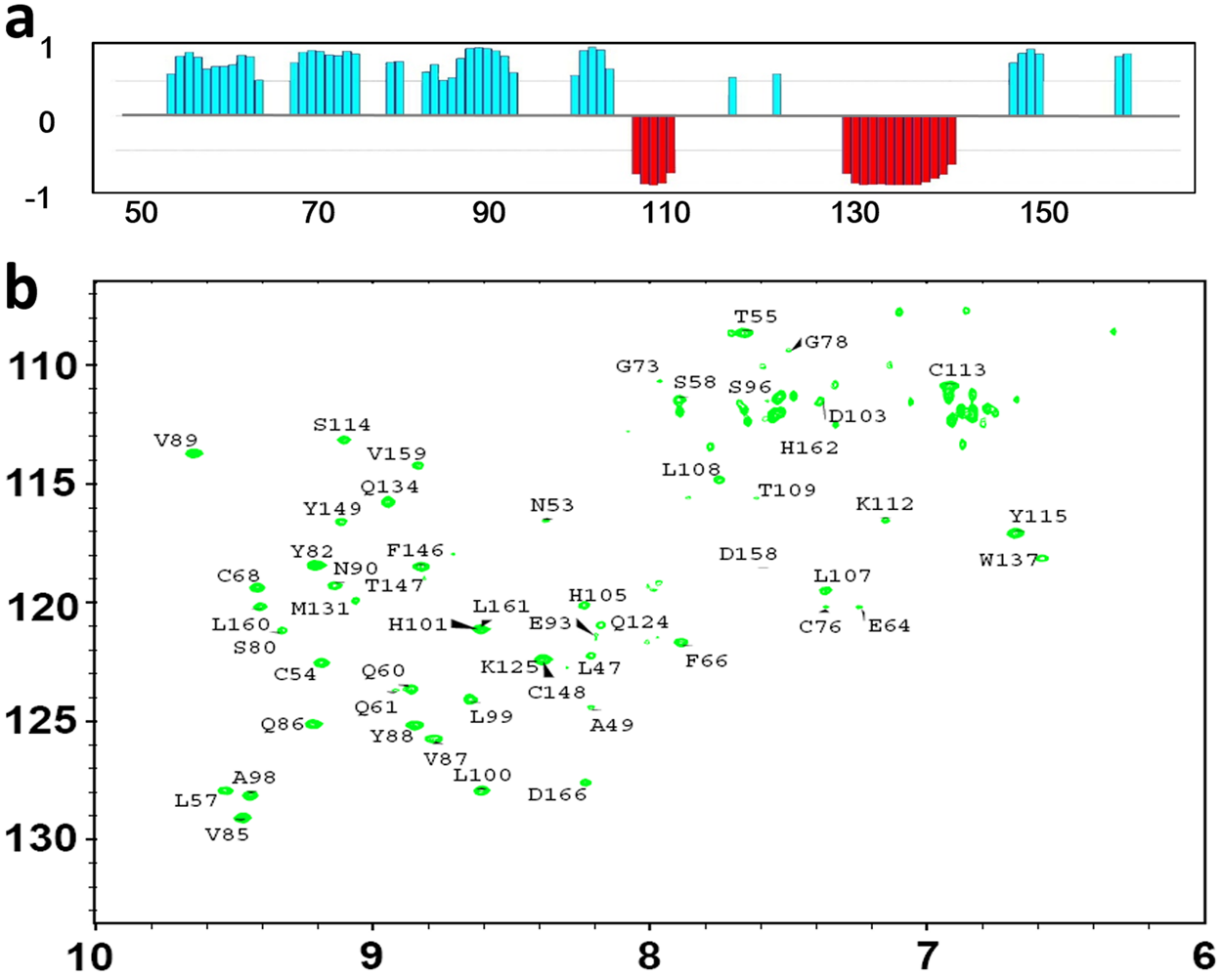
NMR analysis of secondary structure of hβ4-loop. (**a**) The chemical shift index (CSI) generated from TALOS program reveals the secondary structure of hβ4-loop. (**b**) The ^1^H-^15^N HSQC spectrum (after the ^15^N-labeled hβ4-loopsample in H_2_O NMR buffer was exchanged into D_2_O NMR buffer) indicates the H-bond formed in α-helices and β-sheets in hβ4-loop, assignments of the cross-peaks belonging to the corresponding residues were displayed.

**Figure 4:**
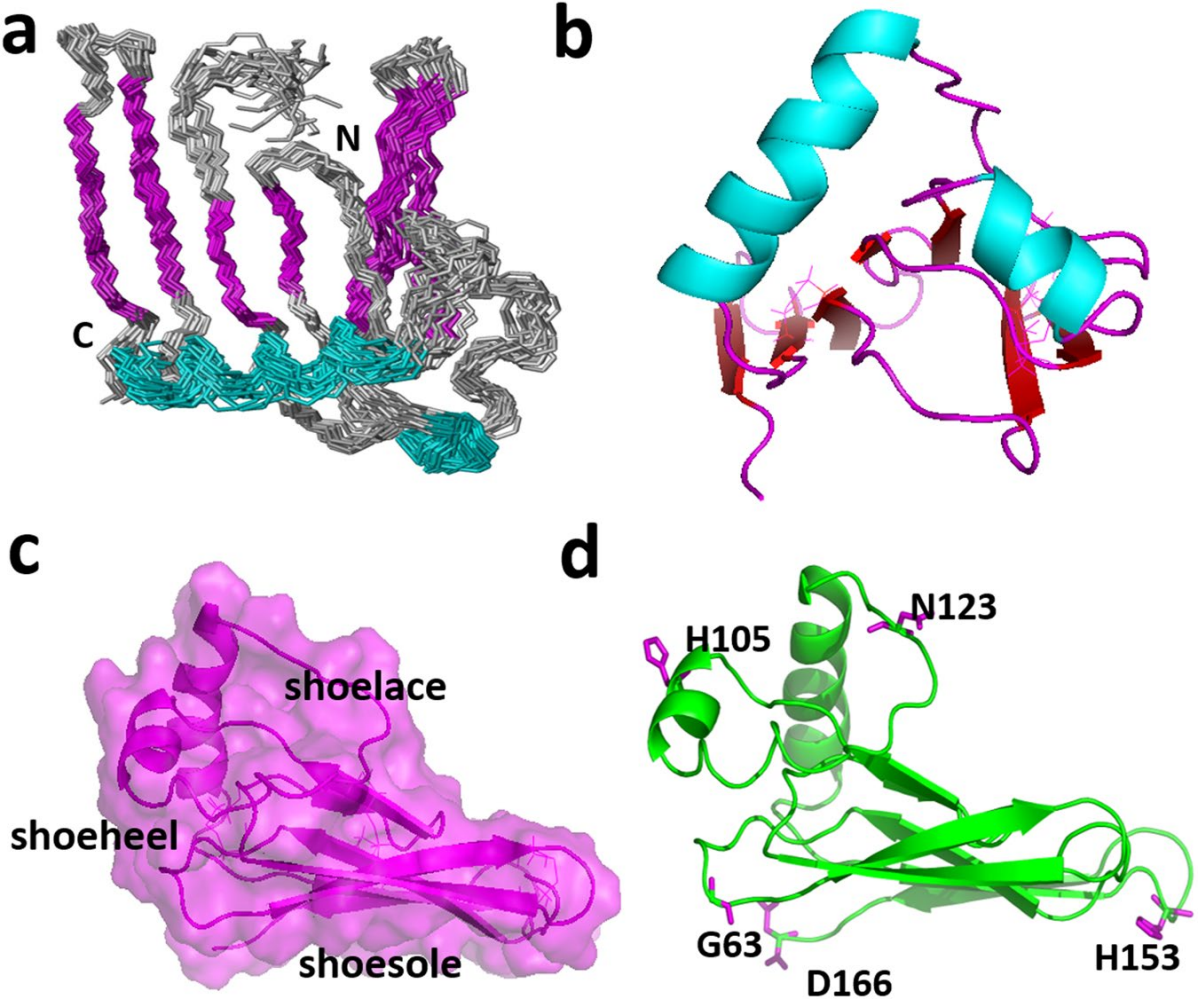
The hβ4-loop folding and its interactions with BKα subunit. (**a**) The ensemble of 20 lowest-energy structures superposed in the secondary regions was displayed in backbone mode. The N- and C-termini were labeled, and the α-helices and β-sheets were colored in cyan and pink, respectively. (**b**) The orientations of the two α-helices in hβ4-loop structure. Helices α1 and α2 were displayed in cyan. (**c**) The shoe-like shape of hβ4-loop global folding displayed in transparent surface and ribbon modes in pink. (**d**) The positions of the residues (Gly63, His105, Asn123, His153 and Asp166, displayed in stick mode in pink) in hβ4-loop with obvious chemical shift perturbation in ^1^H-^15^N HSQC spectra upon being added extracellular peptides of BKα subunit, described in the manuscript.

Then, we tried to determine the 3D solution structure of hβ4-loop by common multidimensional heteronuclear NMR spectroscopies. The secondary structure of hβ4-loop was firstly determined by the chemical shift index generated by program TALOS (Fig. 3a). Then, the H-bonds within secondary structures were confirmed by running H-D exchange ^1^H-^15^N HSQC experiments (Fig. 3b). The conformations of anti-parallel β-sheets were testified by classic NOE patterns observed between them (Figure S2). Finally, in total, 2277 distance restraints from NOE, 80 hydrogen bonds and 166 dihedral angle restraints for backbone φ and ψ angles were used to calculate solution structure (Table 1). A best-fit superposition of the ensemble of the 20 lowest-energy structures (Fig. 4a) was displayed with the RMSD values of 1.19 ± 0.20 Å for global backbone atoms and 1.77 ± 0.77 Å for global heavy atoms. The RMSD values were 0.66 ± 0.12 Å for the backbone (N, C^a^ and CO) atoms and 1.08 ± 0.15 Å for all heavy atoms in the well-ordered second structure regions. The *Ramachandran* plot displays 84.4% of the residues in the most-favored regions and 11.10% residues in additionally allowed regions (Table 1), indicating the solution structures are reasonable.

**Table 1:** NMR structural statistics for hβ4-loop.

Parameters	20 structures
***Distance restraints from NOEs***	
Total NOE	2277
Intra-residue (i-j = 0)	815
Sequential (|i-j| = 1)	690
Medium range (1<|i-j|<5)	332
Long range (|i-j|>5)	440
***H-bond pairs restraints***	80
***Dihedral angle restraints*(**ϕ and ψ)	166
***Structural statistics***	
***r.m.s.d versus the mean structure(Å)***	
All backbone atoms	1.19 ± 0.20
All heavy atoms	1.77 ± 0.17
Backbone atoms (secondary structure)	0.66 ± 0.12
Heavy atoms (secondary structure)	1.08 ± 0.15
***r.m.s.d from the experimental restraints***	
NOE distances (Å)	0.027 ± 0.0028
Dihedral angles	0.78 ± 0.052
***RMSD from idealized geometry***	
Bonds (Å)	0.011 ± 0.00004
Angles (°)	0.74 ± 0.0058
Impropers (°)	0.36 ± 0.016
***Ramachandran analysis*** ^***a***^	
Residues in most favored regions	84.4%
Residues in additionally allowed regions	11.1%
Residues in generously allowed regions	3.3%
Residues in disallowed regions	1.1
***Number of bad contacts/100 residues*** ^***b***^	0
***Overall G-factor*** ^***b***^	0.18

^a^The programs PROCHECK and PROCHECK-NMR were used to check the overall quality of the structure and Gly and Pro are excluded from the Ramachandran analysis.

^b^For the PROCHECK statistic, less than 10 bad contacts per 100 residues, and an overall G-factor larger than −0.5 are expected for a good quality structure.

As shown in Figs 2c, 4a and b, the solution structure of hβ4-loop consists of seven anti-parallel β-sheets (β1, β2, β3, β4, β5, β6 and β7), two α-helices (α1 and α2) and eight loops (L1, L2, L3, L4, L5, L6, L7 and L8), which are arranged in the order of β1- L1- β2-L2- β3- β4- L4- β5- L5- α1- L6- α2- L7- β6- L8- β7. The anti-parallel sheets are not exactly in a single flat plane. Especially, anti-parallel β1, β4 and β5 sheets arrange into a flat plane, forming an angle about 30° with another plane constituted by β2, β3, β6 and β7 sheets. On the whole, hβ4-loop adopts a shoe-like folding (Fig. 4c). The anti-parallel sheets act as a shoesole with a length of 41.2 Å and with a width of 23.7 Å. The N-terminus and C-terminus of hβ4-loop locate in the bottom of this part, and connect two transmembrane helices of β4 subunit, respectively, thus this part might mainly interact with cytomembrane. The two α-helices make up the heel of shoe, which comprises two sides of an almost equilateral triangle (Fig. 4c) with a length of 30 Å of the side and a height of 24.2 Å. Loop L6 between two α-helices looks like a shoelace, which is confined by two disulfide bonds between Cys119 in loop L6 and Cys68 in β2-sheet, and between Cys113 in loop L6 and Cys84 in the C-terminus of β3-sheet. In addition, the conformations of anti-parallel β2 and β3 sheets were stabilized by the disulfide bond between Cys72 and Cys76. The conformation of C-terminal β2-sheet is slightly distorted due to this disulfide bond formation.

### Residue Asn123 of β4 subunit regulates BK channel gating characteristics

To probe possible interactions between BKα and β4 subunits, five extracellular peptides (Fig. 1c) of mSlo1 subunit (hereinafter, mSlo1 stands for BKα subunit in mouse, which has a very high sequence identity to human BKα subunit) were mixed with ^15^N labeled hβ4-loop at a mole ratio 1:1 (Figure S3), respectively. All peptides’ titrations result in almost similar, but slight changes in the chemical shifts of the cross-peaks belonging to His105, His153 and Asp166 and changes in the intensities of the cross peaks (become stronger) assigned as Asn123 and Gly63 in ^1^H-^15^N HSQC spectrum (Figs 4d and S3), indicating that these peptides interact weakly with hβ4-loop, and that these residues might be involved in interactions between hβ4 and BKα subunit. In the BK channel, these extracellular segments connect the transmembrane helices of BKα subunit, their conformations are more rigid than those of the individual peptides in solution. That is to say, these small synthetic peptides might not bind to hβ4-loop at a binding affinity similar to that of BKα subunit binding to hβ4-loop. In addition, the S5-S6 linker 1 was ever suggested to consist of the turret loop of BK channel pore region^24^, thus, it’s still meaningful to probe whether or not these residues, or this peptide is involved into association of BKα with β4 subunits.

**Figure 5:**
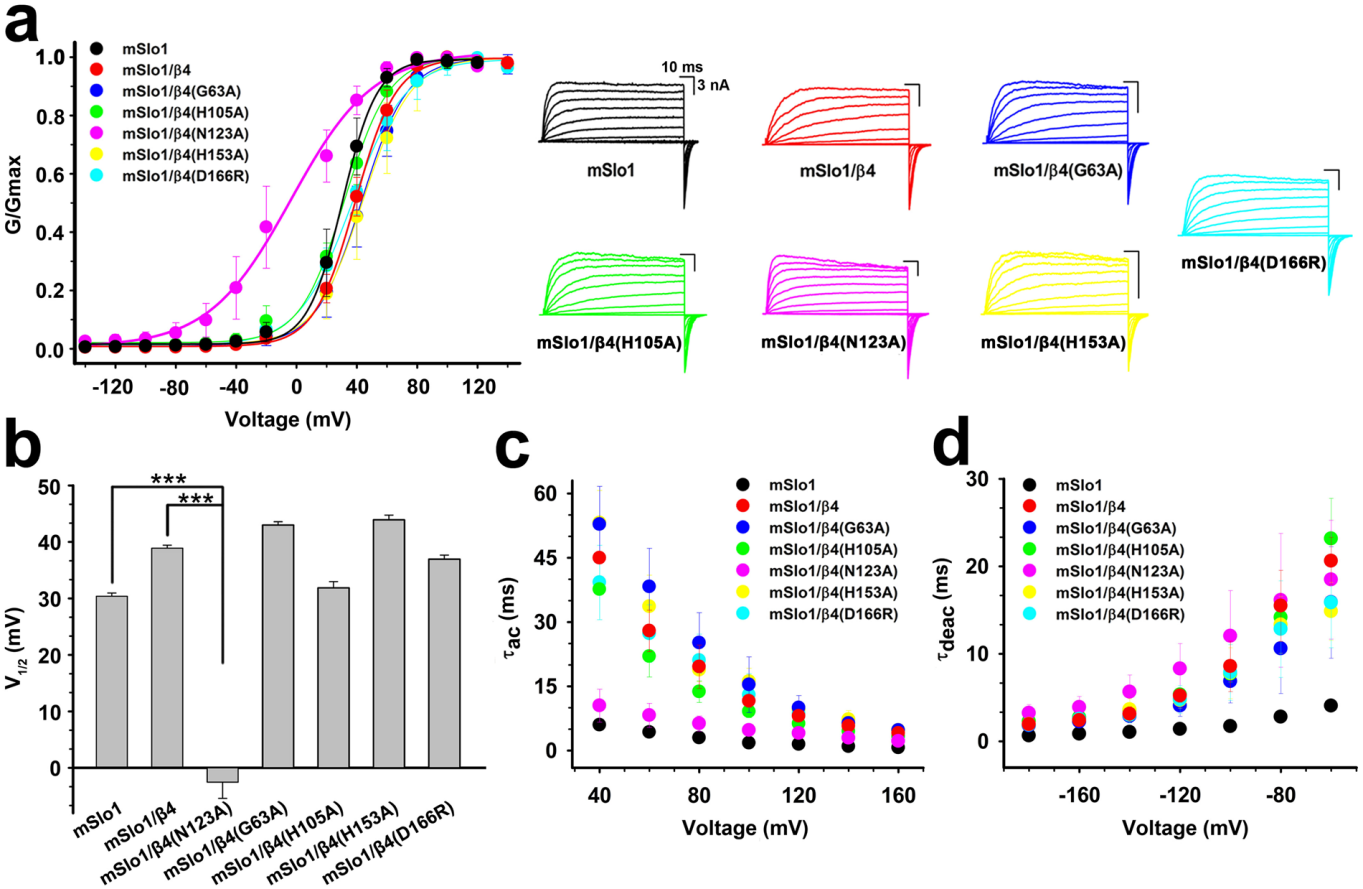
Asn123 of β4 subunit regulates BK channel gating characteristics. (**a**) Left, G-V curves were plotted for mSlo1, mSlo1/β4, mSlo1/β4(G63A), mSlo1/β4(H105A), mSlo1/β4(N123A), mSlo1/β4(H153A) and mSlo1/β4(D166R) channels. Solid lines were single Boltzmann functions fitted to each G-V curve. Right, the representative current traces from inside-out patches under symmetrical 160 mM K^+^ solution by voltage steps from −140 to + 140 mV with an increment of 20 mV in the presence of intracellular 10 μM Ca^2+^. (**b**) the *V*_1/2_ values were 30.39 ± 0.57 mV (n = 7), 38.89 ± 0.53 mV (n = 4), −2.53 ± 2.88 mV (n = 9), 42.99 ± 0.58 mV (n = 5), 31.88 ± 1.11 mV (n = 5), 43.91 ± 0.84 mV (n = 5) and 36.96 ± 0.71 mV (n = 5) for mSlo1, mSlo1/β4, mSlo1/β4(N123A), mSlo1/β4(G63A), mSlo1/β4(H105A), mSlo1/β4(H153A) and mSlo1/β4(D166R) channels, respectively. Data were shown as mean ± S.E., and statistical significance was got using two-tailed unpaired Student’s t-test (***P < 0.001); and (**c**,**d**) mean activation/deactivation time constants (*τ*_ac_ and *τ*_deac_) as functions of membrane voltage were plotted for mSlo1, mSlo1/β4, mSlo1/β4(G63A), mSlo1/β4(H105A), mSlo1/β4(N123A), mSlo1/β4(H153A) and mSlo1/β4(D166R) channels, respectively.

Therefore, these residues were then substituted with alanine in full-length β4 subunit (except that Asp166 was mutated into Arg166), and the effects on BK channel gating characteristics by these mutations were analyzed after co-expression of these β4 mutants with mSlo1 subunit. The G-V curves for the channels mSlo1/β4, mSlo1/β4(G63A), mSlo1/β4 (H105A), mSlo1/β4(H153A) and mSlo1/β4(D166R) are not statistically different from that of the wild-type (WT) mSlo1 alone channel at 10 μM Ca^2+^, whereas β4 mutant N123A activates the channel by significantly shifting the channel G-V curve to more negative voltages (Fig. 5a). The mSlo1/β4(N123A) channel exhibits a change in the slope of G-V curve with respect to the mSlo1 alone channel over a voltage range from −20 mV to + 40 mV, indicating decreased voltage sensitivity. The fractional changes of half activation voltages (*V*_1/2_) of these channels caused by β4 mutants (G63A, H105A, H153A and D166R) are positive, slightly different from that by WT β4, while that caused by β4 mutant N123A is negative (Fig. 5b), implying that the β4 mutation from Asn123 to Ala123 stimulates the channel to traverse into an open state. In addition, the voltage dependence of activation kinetics (*τ*_ac_-V) of BK channel is clearly shifted by β4 mutants G63A, H105A, H153A and D166R in the intracellular 10 μM Ca^2+^ (Fig. 5c), with the effects similar to that by WT β4 subunit^2,7,20,25^. Nevertheless, β4 mutant N123A does not perturb the activation time constant of the channel, its *τ*_ac_-V relationship is almost identical to that of mSlo1 alone channel. However, the changes in deactivation time constant (*τ*_deac_) of BK channel caused by WT β4 subunit and its mutants were almost similar (Fig. 5d), consistent to the fact that β4 subunit distinctly slows BK channel deactivation kinetics. Thus, N123A mutant only makes β4 subunit lose the ability of slowing BK channel activation kinetics. Therefore, in this report we concluded that the residue Asn123 of β4 subunit is critical in regulating BK channel gating characteristics at a given concentration of intracellular 10 μM Ca^2+^.

### Asn123 of β4 subunit regulates the ChTX sensitivity of BK channel

**Figure 6:**
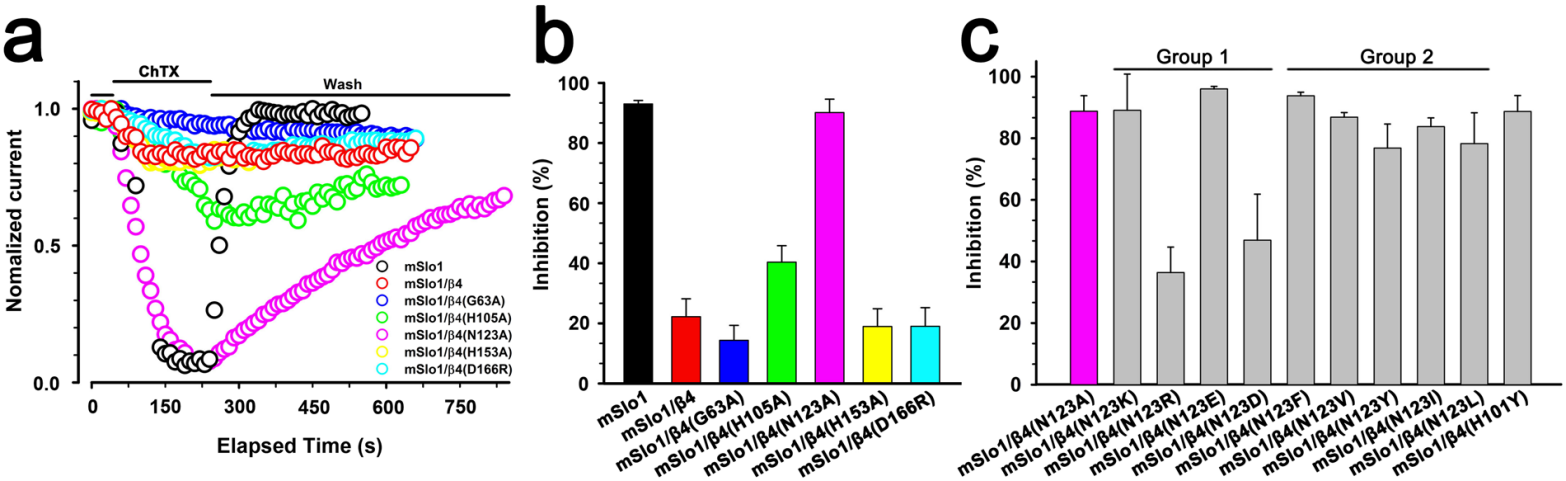
The effects on ChTX sensitivity of BK channel by β4 subunit mutants. (**a**) The time courses of blockade of mSlo1, mSlo1/β4, mSlo1/β4(G63A), mSlo1/β4(H105A), mSlo1/β4(N123A), mSlo1/β4(H153A) and mSlo1/β4(D166R) channels by ChTX. Each patch was perfused with 1 μM ChTX as indicated by the horizontal bars. (**b**) The blockades by 1 μM ChTX were plotted for mSlo1, mSlo1/β4, mSlo1/β4(G63A), mSlo1/β4(H105A), mSlo1/β4(N123A), mSlo1/β4(H153A) and mSlo1/β4(D166R) channels. (**c**) The blockades by 1 μM ChTX were plotted from Slo1/β4(H101Y) and other mSlo1/β4 mutant channels (groups 1 and 2 mutations at Asn123) described in the text.

To further probe whether these residues are related to the ChTX resistance of mSlo1/β4 channel, these β4 mutants were analyzed with respect to ChTX binding after their co-expression with mSlo1 subunit over 1 μM ChTX (Fig. 6a,b). Compared with the currents of mSlo1 channel, the currents of mSlo1/β4 channel were reduced by only about 20% in the amplitude. The on-time course of ChTX blocking the mSlo1/β4 currents was slow (*τ*_on_ is about 60 s), and the off-time course was also much slower than that of mSlo1 channel, indicating that ChTX is confined within a very narrow space. The mSlo1/β4 mutant (G63A, H105A, H153A and D166R) channels display significant resistance to ChTX, slightly different from mSlo1/β4 channel. Notably, the mSlo1/β4(N123A) channel shows about 90% inhibition of currents, almost identical to mSlo1 channel. 1 μM ChTX blocks mSlo1/β4(N123A) channel currents with *τ*_on_ about 72.0 s, and *τ*_off_ about 800 s (Table 2**)**, indicating that mSlo1/β4(N123A) channel has a higher binding affinity (*K*_D_^mSl^°^1/β4(N123A)^ is about 96 nM) to ChTX than mSlo1/β4 channel (*K*_D_^mSl^°^1/β4^ is too weak to be predicted). Therefore, Asn123 of β4 subunit is important in regulating the ChTX sensitivity of BK channel.

**Table 2:** The on-time course (*τ*_on_), off-time course (*τ*_off_) and dissociation constant (*K*_D_) of BK mutant channels determined by fitting a data to a first order bimolecular reaction scheme.

	*τ*_on_ (s)	*τ*_off_(s)	*K*_D_(nM)
mSlo1	3.5 ± 0.4	21.6 ± 0.4	193
mSlo1/β4	60.9 ± 6.7		
mSlo1/β4(G63A)	135.2 ± 28.4		
mSlo1/β4(H105A)	82.4 ± 12.7		
mSlo1/β4(H153A)	36.1 ± 5.6		
mSlo1/β4(D166A)	97.8 ± 14.0		
mSlo1/β4(H101Y)	8.0 ± 0.4	32.6 ± 2.0	325.2
mSlo1/β4(N123A)	72.0 ± 4.9	816.4 ± 19.6	96.8
mSlo1/β4(N123K)	13.3 ± 0.5	313.6 ± 8.1	44.3
mSlo1/β4(N123R)	21.1 ± 2.9	85.3 ± 10.9	328.6
mSlo1/β4(N123E)	10.3 ± 1.4	137.8 ± 9.6	80.8
mSlo1/β4(N123D)	42.2 ± 3.7	623.5 ± 71.0	72.6
mSlo1/β4(N123F)	30.1 ± 1.8	669.8 ± 68.8	47.1
mSlo1/β4(N123V)	3.1 ± 1.0	53.3 ± 2.4	61.8
mSlo1/β4(N123Y)	5.9 ± 0.4	26.2 ± 1.5	290.6
mSlo1/β4(N123I)	4.1 ± 0.5	38.5 ± 5.3	119.2
mSlo1/β4(N123L)	6.9 ± 0.4	171.4 ± 5.6	41.9

**Figure 7:**
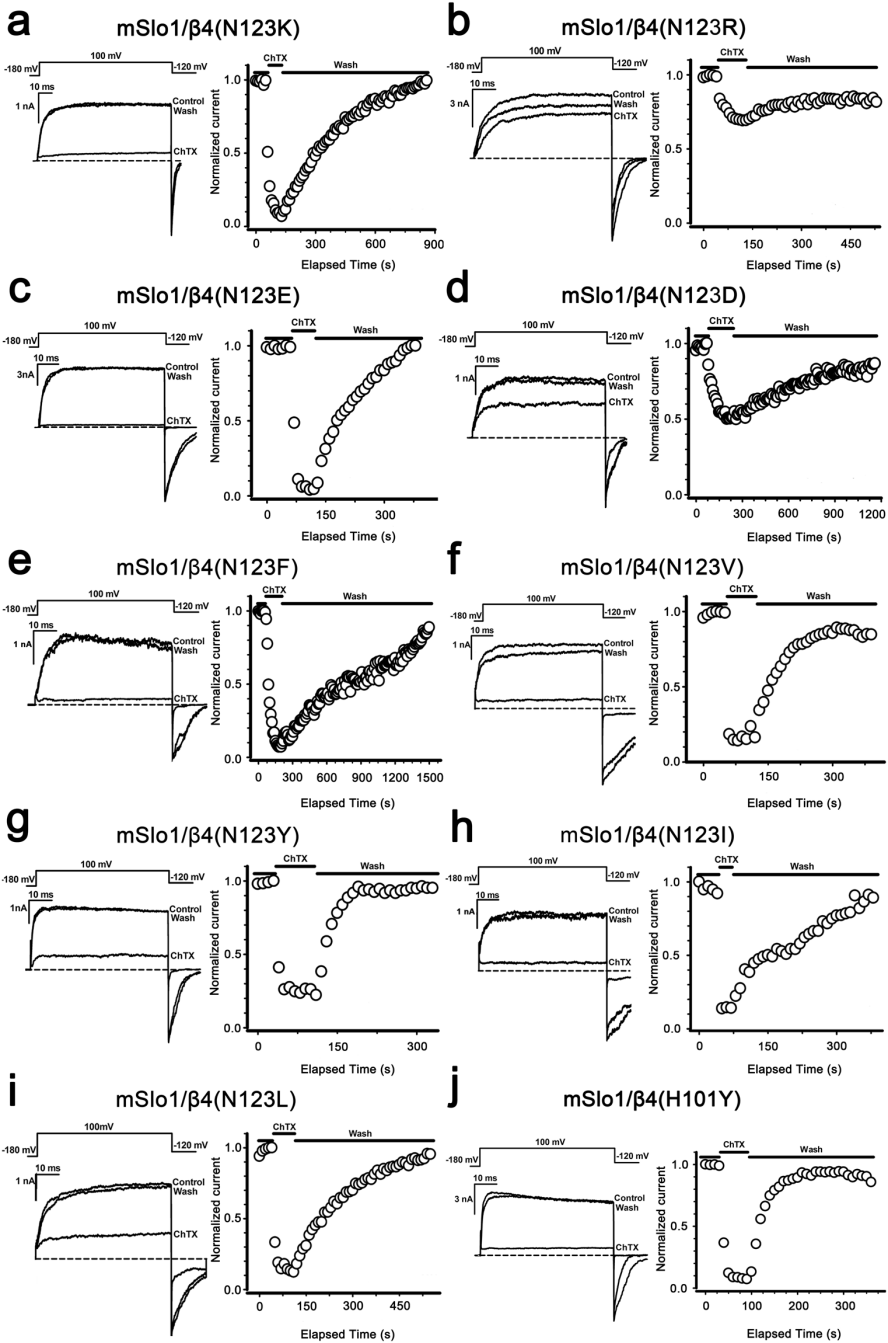
The effect on the ChTX sensitivity of BK channels by the mutations at the positions 123 or 101 of β4 subunit. The traces shown on the left were obtained from outside-out patches from HEK293 cells transfected with cDNA encoding (**a**) mSlo1/β4(N123K), (**b**) mSlo1/β4(N123R), (**c**) mSlo1/β4(N123E), (**d**) mSlo1/β4(N123D), (**e**) mSlo1/β4(N123F), (**f**) mSlo1/β4(N123V), (**g**) mSlo1/β4(N123Y), (**h**) mSlo1/β4(N123I), (**i**) mSlo1/β4(N123L) and (**j**) mSlo1/β4(H101Y) channels, respectively. The representative currents were activated by a voltage step from −180 to +100 mV for 60 ms, in the presence and absence of 1 μM ChTX with intracellular 10 μM Ca^2+^. A voltage protocol was plotted at the top of each trace. Dotted lines represented zero current. On the right panel, the peak currents from the above patches were plotted as a function of elapsed time. Currents were elicited by intracellular 10 μM Ca^2+^ with repetitive voltage steps to +100 mV with a time interval of 10 s, before, during, and after the application of 1 μM ChTX as indicated by the horizontal bars.

In hβ4-loop structure, Asn123 is close to three basic residues (Lys120, Arg121 and Lys125), which were suggested to impede ChTX entry by repulsively electrostatic interaction^20^. To test it, mSlo1/β4 mutant (N123K, N123R, N123E and N123D, group 1 mutations in Fig. 6c) channels were analyzed at 1 μM of ChTX (Fig. 7). As shown in Table 2, all these channels present higher binding affinities to ChTX than mSlo1/β4 channel does. The averaged inhibition values of ChTX on mSlo1/β4, mSlo1/β4(N123K), mSlo1/β4(N123R), mSlo1/β4(N123E) and mSlo1/β4(N123D) channels are 22.22 ± 5.96%, 89.07 ± 11.77%, 36.42 ± 8.25%, 96.01 ± 0.83% and 46.90 ± 14.95%, respectively (Figs 6a and 7a–d). β4 mutants N123R and N123K endow BK channels with ChTX sensitivity at different levels, as N123E and N123D do. Therefore, the inhibition of ChTX on mSlo1/β4 channel is not completely dependent on the electrostatic interactions between β4 subunit and ChTX, which accords with the facts that ChTX interacts with the inserted hydrophobic residue Tyr100 (inY100) in β4 subunit^20^, or Tyr101 in our β4 H101Y mutant (Figs 6c and 7). The faster on-time course of mSlo1/β4(H101Y) channel (*τ*_on_ = 8 s) than that of mSlo1/β4 channel implies that Tyr101 is accessible for ChTX. We further replaced Asn123 with hydrophobic residues (Phe, Tyr, Leu, Val and Ile, group 2 mutations in Fig. 6c) and examined their effects with the application of 1 μM ChTX (Figs 6a and 7e–i). These mSlo1/β4 mutant channels also display higher binding affinities to ChTX than mSlo1/β4 channel. The inhibition extents of ChTX on these mSlo1/β4 mutant channels are almost identical to that on mSlo1 channel, suggesting that the hydrophobic modification at Asn123 in β4 subunit remarkably increases the sensitivity of BK channels to ChTX. Their *τ*_on_ or *τ*_off_ values are slower than those of mSlo1 alone channel, revealing that ChTX blocking these mutant channels is still confined in a limited space, and that mSlo1 is still associated with β4 mutants.

### Glu264 in the BKα subunit might interacts with Asn123 in β4 subunit

**Figure 8:**
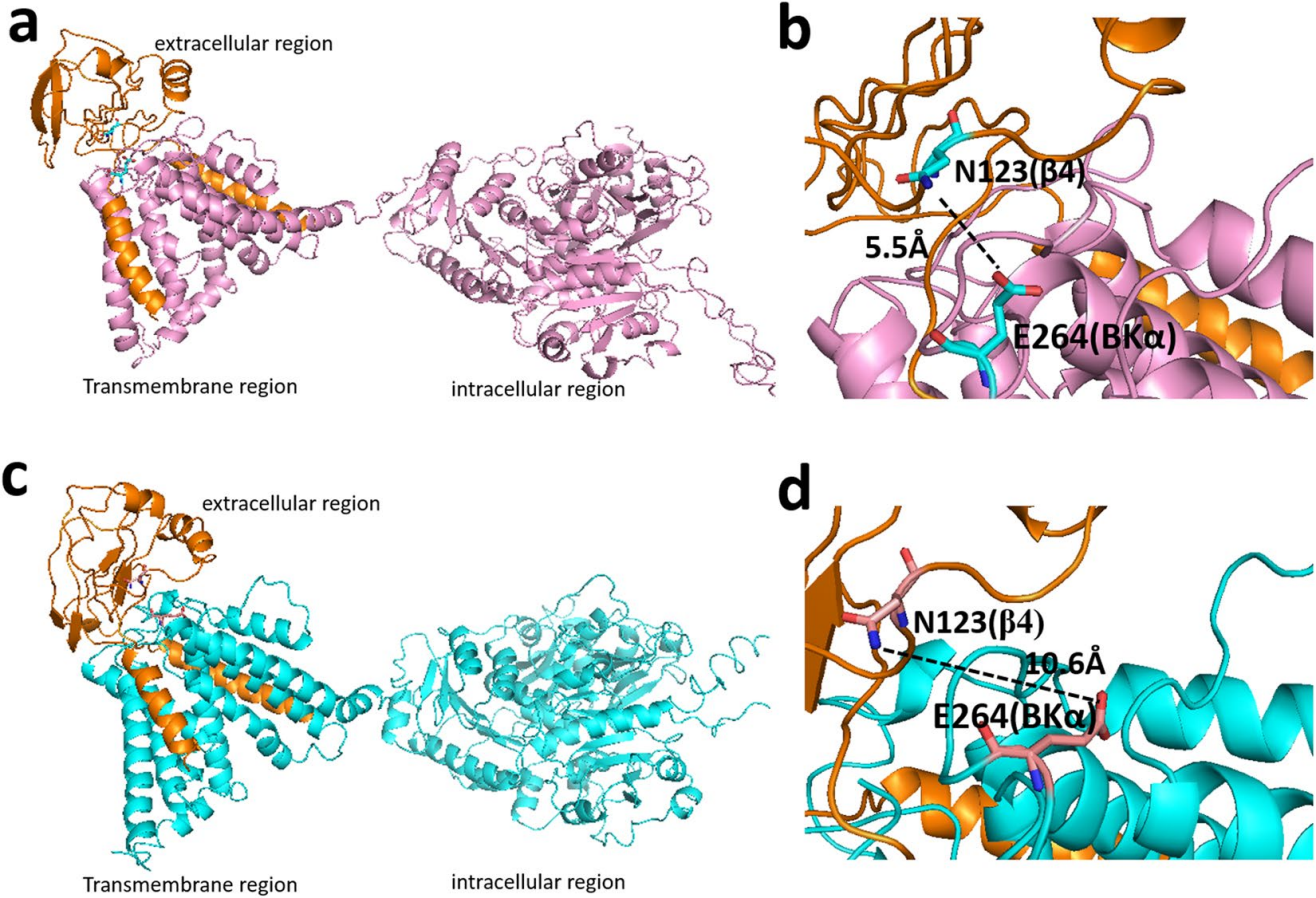
The interaction between full-length β4 subunit and BKα subunit in the structural models constructed using cryo-EM structures of full-length Slo1 channel from *Aplysia californica* (**a**) in the presence of Ca^2+^ (pdb code 5TJ6) and (**c**) in the absence of Ca^2+^ (pdb code 5TJI), respectively. (**b** and **d**) The distance between the sidechains of Asn123(β4) and Glu264(BKα) from structural models in (**a**) and (**c**). The extracelluar region, transmembrane region and intracellular region of the two structural models were labeled in (**a**) and (**c**). In all figures, human BKα subunit (displayed in pink (**a**) and (**b**), in cyan in (**c**) and (**d**)) and β4 subunit (displayed in orange) were in ribbon mode. In (**b**) and (**d**), the residues Asn123(β4) and Glu264(BKα) were displayed in stick mode. The dotted lines show distances between the sidechains of these two residues.

Given the observations above, we inferred that Asn123 might directly interact with certain residue in BKα subunit. In hβ4-loop, residues Lys120, Arg121 and Lys125 were thought close to BK channel pore region^20^. Previously, residues Glu257, Asp261 and Glu264 in S5-S6 linker 1 of mSlo1 subunit were suggested to interact with β4 Lys120, Arg121 and Lys125^20^. But, so far, no direct evidences were presented to testify this hypothesis. We therefore assume that it is not Lys120, Arg121 and Lys125, but Asn123 of β4 subunit that may interact with these residues of mSlo1. Therefore, we constructed structural models of human BKα/β4 in complex with ChTX using the recently reported two cryo-EM structures (pdb codes 5TJI and 5TJ6) of BK channel from *Aplysia californica* (with sequence identity 56% to that of human BKα, Figure S4) in the absence of and in the presence of Ca^2+ ^^26,27^, respectively (Figs 8, S5a and S5d). In both models, the ChTX locates in the center of the β4 tetramer (Figure S5b and S5e). Four pairs of Asn123(β4)-Glu(BKα) are around ChTX (Figures S5c and S5f). In the structural model calculated based on the structure of BKα channel in the presence of Ca^2+^, Asn123(β4) was found to be close to Glu264(BKα) with a distance of 5.5 Å between their side-chains, indicating that Asn123(β4) might interact with Glu264(BKα).

**Figure 9:**
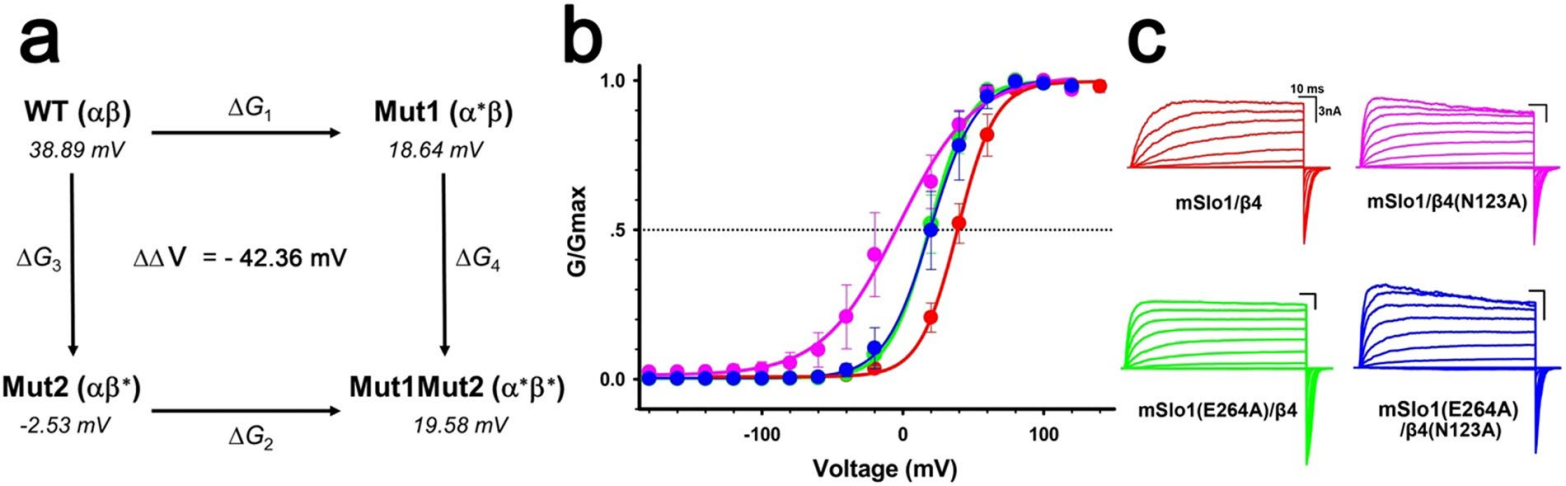
Possible interaction between Asn123(β4) and Glu264(α) determined by double-mutant cycle analysis. (**a**) Representative double-mutant cycle for mSlo1(E264A) and β4(N123A) shows a significant energy coupling. The *V*_1/2_ values of the WT and each mutant were from the Boltzmann functions fitted G-V curves at 10 μM Ca^2+^. The measured *V*_1/2_ values for each channel are shown in *italic*s. (**b**) G–V curves plotted for mSlo1/β4 (*V*_1/2_ = 38.89 mV, n = 4, red), mSlo1/β4(N123A) (*V*_1/2_ = −2.53 mV, n = 9, pink), mSlo1(E264A)/β4 (*V*_1/2_ = 18.64 mV, n = 6, green), mSlo1(E264A)/β4(N123A) (*V*_1/2_ = 19.58 mV, n = 6, blue). (**c**) Representative currents from each experimental combination, recorded from inside-out patches, activated by voltage steps from −160 mV to +160 mV in increment of 20 mV, after a prepulse of −180 mV, in 10 μM Ca^2+^.

To test their possible interaction, the thermodynamic double-mutant cycle analysis^28–30^ was employed. Generally, this analysis can quantify the influence of one mutation on the effect of the second mutation through a pairwise coupling energy between the two mutated residues. As depicted in Fig. 9a, a representative double-mutant cycle involves WT complex (αβ), two single mutants (α^*^β and αβ^*^) and the corresponding double mutant (α^*^β^*^)^6,30,31^. Here ^*^ denotes a mutation. The free energy of coupling between two mutated residues (ΔΔ*G*_int_) can be calculated using the equation ΔΔ*G*_int_ = Δ*G*_1_ − Δ*G*_2_ = Δ*G*_3_ − Δ*G*_4_^31^. If the total free energy change ΔΔ*G*_int_ shows a significant change (the absolute value is ≥1 kcal/mol), the pairwise residues on α and β subunits would be judged to be coupled^32^. Considering that the free energy of a channel is in direct proportion to *V*_1/2_, we can measure the corresponding *V*_1/2_ instead. Thus, this method can be described as the following equation: ΔΔ*V*_1/2_ = Δ*V*_1/2 (αβ*)_ + Δ*V*_1/2 (α*β)_ − Δ*V*_1/2 (α*β*)_ − Δ*V*_1/2 (αβ)_. A distinct change of ΔΔ*V*_1/2_ ≥ 20 mV (or 1 kcal/mol) would be judged to be coupled. To minimize the possibility of new interactions being formed, interacting residues are usually replaced with Ala^33^. Thus, Asn123 in β4 subunit and Glu264 in mSlo1 were substituted with Ala and their G-V curves with the corresponding currents of mSlo1(E264) versus β4(N123) cycle were shown in Fig. 9b,c. The double mutant mSlo1(E264A)/β4(N123A) is displaced along the voltage axis by an amount roughly equal to the single mutant mSlo1(E264A). The Glu264(mSlo1) and Asn123(β4) pair shows obvious energetic coupling, revealed by the change of ΔΔV (−42.36 mV) value, calculated from the G-V curves of the four channels comprising the double-mutant cycle at 10 μM Ca^2+^. Thus, Asn123 in β4 subunit might interact with Glu264 of BKα subunit through hydrogen-bond based on the properties of their side-chains in nature.

## Discussion

In hβ4-loop structure, Asn123 locates in the loop L6, which is adjacent to the pore of the channel. The mutations from Asn123 to hydrophobic residues, such as Ala123, Val123, Ile123, Phe123, Tyr123, and Leu123, damage the hydrogen bond interaction between Asn123 of β4 subunit and Glu264 of BKα subunit, which further forces the side-chains of residue 123 to extend into the narrow space where ChTX is. ChTX contains 6 basic residues (Lys11, Arg19, Arg25, Lys27, Lys31 and Lys32) and 4 hydrophobic residues (Phe2, Val5, Trp14 and Val16), locating dispersedly in the peptide, in favor of hydrophobic or electrostatic interactions of ChTX with hβ4-loop. The faster on-time courses on ChTX sensitivity of the mSlo1/β4 mutant (including N123V, N123L, N123I, N123F and N123Y) channels (*τ*_on_ values are equal to or smaller than 30 s) than that of mSlo1/β4 channel (*τ*_on_ value is about 60 s) indicate that the more the hydrophobicity of residue 123, the more accessible the site for ChTX. The mSlo1/β4 mutant (N123A, N123L, N123F, N123I, N123V) channels therefore demonstrate higher binding affinities to ChTX than mSlo1/β4 channel (Table 2). However, due to the limited space where ChTX is and the bigger size of side-chain of residue Tyr than that of residue Phe, mSlo1/β4(N123Y) and mSlo1/β4(H101Y) channels bind to ChTX much weaker than mSlo1/β4(N123F) channel.

When Asn123 is replaced by positively charged residues Arg123 or Lys123, or by negatively charged residues Glu123 or Asp123, mSlo1/β4(N123R) channel displays resistance to ChTX almost similar to mSlo1/β4 channel, while mSlo1/β4(N123K), mSlo1/β4(N123D) and mSlo1/β4(N123E) channels present resistance to ChTX at different levels, but are weaker than that of mSlo1 channel. To explain differences in the behavior of ChTX blockade of these mSlo1/β4 mutant channels, we analyzed the assembled structural models of BKα/β4 channel in complex with ChTX (Figure S5). In these two models, four pairs of Glu264 (BKα subunit)-Asn123 (β4 subunit) are around ChTX. The electrostatic potential analysis indicates that the interaction between ChTX and β4 subunit is not completely pure electrostatic characteristics, as described in the previous report^20^. Upon Asn123 being replaced by Arg123 or Lys123, the interactions between mSlo1 and β4 subunits are enhanced. However, the sidechain of Arg is larger than that of Lys, which may strengthen the electrostatic repulsion between β4-loop and ChTX in the narrow space, thus mSlo1/β4(N123R) channel binds ChTX weaker than mSlo1/β4(N123K) channel. On the contrary, the mutations from Asn123 to Asp123 and Glu123 weaken the interactions between mSlo1 and β4 subunits, but probably strengthen the interactions between ChTX and β4 subunit through ionic bonds or hydrogen-bonds. Similarly, Glu123 with a little longer side-chain than Asp123 also favors its hydrophobic interaction with ChTX, which results in higher binding affinity of mSlo1/β4(N123E) channel to ChTX than that of mSlo1/β4(N123D) channel.

Among the four β subunit family members, only the brain-enriched hβ4 shows extremely low sensitivity to the ChTX^1^. The extracellular loop of β subunits plays an important role in determining the characteristics of toxin binding^34^. When the extracellular loops of the β1 and β4 subunits are exchanged, the phenotypes obtained regarding toxin binding correspond to the respective loops^34^. Furthermore, the non-glycosylated β4 is less effective than WT β4 in protecting the channel against toxin block^17^. This fact indicates that the complex oligosaccharide chains on the extracellular loop of β4 may compete with toxin accessibility to the channel pore. In addition, the β4 subunit reduces the voltage sensitivity of BK channel activation and has complex effects on apparent Ca^2+^ sensitivity^12^, presenting a mechanism underlying β4 subunit action in the context of a dual allosteric model for BK channel gating. In current work, the solution structure of the extracellular loop of β4 will accelerate our understanding of the molecular mechanism for ChTX resistance of BK channel conferred by β4 subunit. Using electrophysiology, we also found that the mutations on the residue Asn123 of β4 subunit affected BK channel G-V curve, activation time constant at 10 μM Ca^2+^. The present results indicate that the β4 subunit extracellular loop may play a role in the modulation of the channel gating characteristic. We also calculated the energetic coupling between Asn123(β4) and Glu264(α) through molecular modeling and thermodynamic double-mutant cycle analysis, suggesting that Asn123(β4) might interact with Glu264(α). The residues Asn123(β4) and Glu264(α) are conserved among human, mouse, rabbit and monkey (Figures S6 and S7), this functional data thus may be applicable in other hosts. These studies may be helpful to design new drugs specifically targeted BKα/β4 channel subtypes.

## Experimental Procedures

### Cloning, protein expression and purification

DNA encoding human β4 (KCNMB4, GenBank accession no. AF207992.1) extracellular loop (45–166 aa) was cloned into a pETDuet expression vector (Novagen) with a His_6_ tag and an MBP tag at N-terminus, as described previously^35^. Origami BL(DE3) (Novagen) cells were transformed with the vector, and grown in M9 minimal medium containing ^13^C-labeled D-glucose and ^15^NH_4_Cl as the sole carbon and nitrogen source, respectively. The protein expression was induced with 0.5 mM IPTG at 18 °C for 20 hours. The cells were lysed by sonication in lysis buffer (25 mM Tris, 500 mM NaCl, pH 7.5). The insoluble fraction was removed by centrifugation (16,000 g) for 20 min at 4 °C. The soluble fraction was loaded into a Ni^2+^ sepharose resin column (Roche), washed a solution comprising 25 mM Tris, pH 7.5, 500 mM NaCl, 30 mM imidazole, and eluted with an imidazole gradient of 30–500 mM. His_8_-MBP tag was removed by thrombin cleavage. Then, the solution was concentrated, passed through an amylose resin chromatography column pre-equilibrated with 25 mM Tris, pH 7.5, 150 mM NaCl and 0.5 mM EDTA, and subsequently purified on a Hiload 16/600 Superdex 75 column (GE Healthcare, USA), washed with the buffer containing 25 mM NaH_2_PO_4_, pH 6.80 and 50 mM NaCl.

For NMR titration experiments, based on the topological structure of mSlo1 (KCNMA1, GenBank accession No. HQ221747.1), five extracellular peptides (Fig. 1c) of mSlo1were commercially synthesized at an HPLC grade, confirmed by mass spectrometry, from GL Biochem Ltd (Shanghai, China), termed as NH_2_, S1-S2 linker (*i.e*., the linker between S1 and S2 transmembrane helices), S3-S4 linker (*i.e*., the linker between S3 and S4 transmembrane helices), S5-S6 linker 1 and linker 2 (*i.e*., the linker between S5 and S6 transmembrane helices), respectively. The topology of BKα subunit and β4 subunit, and the peptides selected for NMR titration experiments were displayed in Fig. 1.

### Mass spectroscopy for disulfide bond determination

The agents such as acetonitrile, formic acid and acetic acid were purchased from JT Baker, trypsin was purchased from Promega, chymotrypsin was purchased from Roche Ltd (Germany), N-ethylmaleimide (NEM), tris-(2-carboxyethyl)-phosphine (TCEP) and other general chemicals were bought from Sigma-Aldrich, respectively. For protein molecular weight measurement, protein sample was diluted with 0.1 M acetic acid, pH 3.0 to 1 μg/μL. 50 μL of protein solution was incubated in the presence of or in the absence of 10 mM TCEP at 65 °C for 10 min, as the reduced form and native form, respectively. 2 μg of protein samples was analyzed on the EASY-nLC1000 HPLC system (Thermo Fisher Scientific) using a self-packed column (75 μm × 70 mm; 3 μm ReproSilPur C4 beads, 300 Å, Dr. Maisch GmbH, Ammerbuch, Germany) at a flow rate of 300 nL/min using 25 min gradients. Mass spectroscopy data were acquired on an LTQ Velos Pro-Orbitrap Elite platform (Thermo Fisher Scientific). The full mass (400–1800 m/z) was scanned in the Orbitrap analyzer with R = 60,000 (defined at m/z 200).

Accurate disulfide bond determination was performed as previously described with slight modifications^21^. 100 μg of protein sample was diluted with a buffer of 0.1 M acetic acid, 4 mM NEM, pH 3.0, loaded on a 10 KDa Microcon filtration devices (Millipore) and centrifuged at 14,000 g for 40 min at 20 °C. Then, the concentrates were diluted in the devices with 0.2 mL of 4 mM NEM buffered by 100 mM Tris, pH 6.5 and centrifuged twice. Finally, 100 μL of 100 mM Tris and Trypsin or Chymotrypsin (1:25, enzyme vs protein) was added to the sample, which was then incubated at 37 °C for 16 hrs. The tryptic peptide mixtures were collected and dried using Speed-Vac. The mass spectrometry analysis was performed on the LTQ Velos Pro-Orbitrap Elite (Thermo Fisher Scientific) platform as previously described^21^. The raw data was searched against hβ4-loop protein sequence database using pLink software^36^. The parameters for pLink search were listed as follows: three missed cleavage sites for trypsin/chymotrypsin per chain, peptide length 4–100 aa, cross-linker disulfide −2.01565 Da on cysteine. The pLink search results were filtered by requiring ≤10 p.p.m. deviation in the observed precursor mass from the mono- isotopic or the first, second, third or fourth isotopic mass of the matched candidate. Candidate disulfide-linked peptides were filtered with an *E*-value cutoff of 0.01; the inter-peptide disulfide bonds were manually checked with following filtering criteria: the two chains contain at least three continuous b or y series ions, and major peaks were assigned to expected ions. The final results from this section were shown Figure S1.

### NMR spectroscopy and analysis

The NMR samples contain about 0.5 mM uniformly ^13^C/^15^N-labelled hβ4-loop in NMR buffer (25 mM NaH_2_PO_4_, pH 6.80, 50 mM NaCl, 0.01% NaN_3_ and 10% D_2_O). All NMR experiments were performed at 20 °C on a Varian Unity Inova 600 NMR spectrometer equipped with a triple resonances cryoprobe and pulsed field gradients. The standard suite of experiments for assigning the ^1^H, ^13^C and ^15^N backbone and side chain chemical shifts of β4-loop and collecting the NOE-based distance restraints were measured, including the 2D ^13^C-edited HSQC and ^15^N-edited HSQC; the 3D HNCA, HNCO, HNCACB, CBCA(CO)NH, HBHA(CO)NH, HCCH-TOCSY, HCCH-COSY, ^15^N-resolved HSQC-TOCSY, ^15^N-resolved HSQC-NOESY, ^13^C-resolved HSQC-NOESY for both aliphatic and aromatic resonances. Spectra were processed with software NMRPipe^22^ and analyzed with program Sparky 3 (http://www.cgl.ucsf.edu/home/sparky/).

To investigate the possible interactions between hβ4-loop and the extracellular peptides in BKαsubunit, NMR titration experiments were performed. The ^15^N-labelled hβ4-loop and the titrants (different peptides) were mixed at a 1:1 molar ratio of hβ4-loop: peptide in NMR buffer (25 mM NaH_2_PO_4_, pH 6.80, 50 mM NaCl and 10% D_2_O). The ^1^H-^15^N HSQC spectra of hβ4-loop were collected after each addition. The NMR titration results were displayed in Figs 4d and S3. To confirm H-bonds formed in the secondary structures, the H-D exchange ^1^H-^15^N HSQC experiments were also performed after the ^15^N-labeled hβ4-loopsamplein H_2_O NMR buffer was exchanged into D_2_O NMR buffer through dialysis.

### NMR structure determination

Structural calculation was performed using a standard simulated annealing protocol implemented in the XPLOR-2.37 program (NIH version)^37^. The inter-proton distance restraints derived from the NOE intensities were grouped into three distance ranges, namely 1.8–2.9 Å, 1.8–3.5 Å and 1.8–6.0 Å, which corresponds to strong, medium and weak NOEs, respectively. The dihedral angles phi and psi were derived from the backbone chemical shifts (HN, HA, CO, CA) by using the program TALOS^23^. A total of ten iterations (50 structures in the initial eight iterations) were performed. 100 structures were computed in the last two iterations, 20 conformers with the lowest energy were used to represent the 3D structures (Fig. 4a). The conformers of the bundle do not violate the following constraints: NOE >0.3 Å and dihedral angle >3°. The entire structure statistics for them were evaluated with PROCHECK and PROCHECK-NMR^38^ and summarized in Table 1. All of the structure figures were generated using the PyMOL (http://pymol.org/) or MOLMOL programs. The structural analysis was displayed in Fig. 4.

#### Pseudo full-length BKα-hβ4 complex assembly

Since NMR structure of hβ4-loop is available, the *pseudo* full-length BKα/hβ4 complex can be modeled by *in silico* method. First, the partial model of β4 subunit was constructed by connecting NMR-determined hβ4-loop structure and two transmembrane helices. The tetrameric conformations of hSlo1 subunit was built using the latest cryo-EM structures of the high conductance Ca^2+^ activated K^+^ channel (PDB codes 5TJ6 and 5TJI)^26,27^ as homology templates with highly overall sequence similarity 66.4, using the program Modeller 9.18 (https://salilab.org/modeller/release.html). BKα interacts with β4 at mole ratio of 1:1, the complex of BKα and β4 was then assembled manually^39–41^. The S0, S1, S2 helix of BKα and the TM1 and TM2 of β4 was orientated according to the previous reports^41,42^. Due to their flexibility, the N-terminal loop (with a sequence of M_1_DALIIPVTM E_11_VPCDSRGQR_20_) and the long loops, with the amino acid sequences of P_622_KRIKKCGCK R_632_PKMSIYKRM R_642_RACCFDCGR S_652_ERDCSCMSG R_662_VRGNVDTLE R_672_AFPLSSVSV N_682_DCSTSFRAF E_692_DEQPSTLSP K_702_KKQRNGGMR N_712_SPNTSPKLM R_722_HDPLLIPGN D_732_QIDNMDSNV K_742_KYDST_747_ and H_55_CGGKTKEAQ K_65_INNGSSQAD G_75_TLKPVDEKE E_85_AVAAE_90_, were deleted during construction of both structural models. Finally, the charybdotoxin (ChTX) was manually docked into the outside of BK pores according to two known structures with PDB codes 4JTA and 4JTC^43^. The final complex models of BKα/β4 was refined by Amber14 with ff14SB force field in generalized born implicit solvent (GBIS). After 20000 steps minimization for the coarse complexes, followed by 1,000,000 heating steps for 0 K to 325 K, the ensembles were finally under 50,000,000 steps equilibrations^44^. Final stable complex models were extracted from the trajectories of production simulations and analyzed in detail as general, displayed in Figures S5.

### Electrophysiological study

For electrophysiological experiments, the full-length cDNAs for mSlo1 and hβ4 were cloned into pcDNA3.1(+) vector (Invitrogen). Mutations were created with the Quick-Change site-directed mutagenesis kit (Stratagene). All of the constructs were verified by DNA sequencing. HEK293 cells were cultured and transiently transfected as reported^20^. Electrophysiological experiments were performed at 1–2 days after transfection. For recordings, all experiments were carried out with excised patches, including inside-out and outside-out recording configuration. During inside-out patches, 10 μM Ca^2+^ solution was perfused locally into cells via a perfusion pipette containing seven solution channels. For outside-out recording, the pipette intracellular solution contained 160 mM MeSO_3_K, 10 mM HEPES, 5 mM HEDTA with added Ca^2+^ to make 10 μM free Ca^2+^, as defined by the EGTAETC program (McCleskey, Vollum Institute, Portland) at pH 7.0. The extracellular solution included 160 mM MeSO_3_K, 10 mM HEPES, pH 7.0, 2 mM MgCl_2_ titrated with MeSO_3_H. The ChTX solution was made by adding 1 mM ChTX (Sigma-Aldrich, Art. No. C7802) to the extracellular solution.

Electrophysiological experiments were performed using a PC2C patch clamp amplifier with corresponding software (InBio, China). Currents typically were digitized at 100 kHz and filtered at 5 kHz. Recorded data were analyzed using Clampfit (Axon Instruments, Inc.), Sigmaplot (SPSS, Inc.) software. Unless otherwise stated, the data were presented as mean ± S.D. The conductance-voltage (G-V) curves for activation were generated from steady-state currents, converted to conductance, and then fitted by the Boltzmann function with the form as follows, G/G_max_ = (1 + exp((*V* − *V*_1/2_)/*κ*))^−1^, where *V*_1/2_ is the voltage at which the conductance (G) is half the maximal conductance (G_max_) and *κ* is a factor affecting the steepness of the activation. The time courses of onset (*τ*_on_) and offset (*τ*_off_) were obtained by fitting data into a first order bimolecular reaction scheme. Data points were evaluated over two-time regimes where *t*_0_ = 0 is the time of drug application and *t*_1_ is the time of drug washout. During application of drug (for *t*_0_ < *t* < *t*_1_), the *τ*_on_ value was determined according to *I*(*t*) = (*I*_0_ − *I*_ss_) * exp(−*t*/*τ*_on_) + *I*_ss_ with *I*_0_, mean control current amplitude before drug application; and *I*_ss_, steady-state current at a given toxin concentration. During recovery (for *t* > *t*_1_), the *τ*_off_ was determined by a fit of the blocking reaction according to *I*(*t*) = *I*_r_ − (*I*_r_ − *I*_ss_) *exp(−(*t* − *t*_1_)/*τ*_off_) with *I*_r_, residual current before washout. The equilibrium dissociation constant (*K*_D_) was defined by *K*_D_ = [ChTX]/((*τ*_off_/*τ*_on_) − 1)^20,45^. The final results from electrophysiological experiments were shown in Figs 5–7 and 9 and Table 2.

### Accession codes

The coordinates of NMR structure of hβ4-loop had been deposited with RCSB Protein Data Bank under accession number 5Y7L. Its chemical shift assignments were also deposited with BMRB ADIT-NMR online deposition system under the accession number 36113.

## Electronic supplementary material

supplementary information
