# Author Correction: Solution structure of extracellular loop of human β4 subunit of BK channel and its biological implication on ChTX sensitivity

**DOI:** 10.1038/s41598-020-60956-w

**Published:** 2020-02-26

**Authors:** Yanting Wang, Wenxian Lan, Zhenzhen Yan, Jing Gao, Xinlian Liu, Sheng Wang, Xiying Guo, Chunxi Wang, Hu Zhou, Jiuping Ding, Chunyang Cao

**Affiliations:** 1 State Key Laboratory of Bioorganic and Natural Product Chemistry, Center for Excellence in Molecular Synthesis, Shanghai Institute of Organic Chemistry, Chinese Academy of Sciences (CAS), 345 Lingling Road, Shanghai, 200032 China; 2 Key Laboratory of Molecular Biophysics of the Ministry of Education, College of Life Science and Technology, Huazhong University of Science and Technology, 1037 Luoyu Road, Wuhan, 430074 Hubei China; 3 Department of Analytical Chemistry, Shanghai Institute of Materia Medica, CAS, 555 Zuchongzhi Road, Shanghai, 201203 China; 4 University of Chinese Academy of Sciences, No. 19(A) Yuquan Road, Shijingshan District, Beijing, 100049 China; 5 Collaborative Innovation Center of Chemistry for Life Sciences, Chinese Academy of Sciences (CAS), 345 Lingling Road, Shanghai, 200032 China

Correction to: *Scientific Reports* 10.1038/s41598-018-23016-y, published online 15 March 2018

This Article contains a typographical error in the Acknowledgements section.

“This work was supported by National Key R&D Program of China (2016YFA0502302 and 2017YPE0108200), by the Strategic Priority Research Program of the Chinese Academy of Sciences (XDB 20000000), by National Science Foundation of China (NSFC) under No. 91753119, 21472229 and 21778065, by the Fundamental Research Funds for the Central Universities, 2016YXMS261.”

should read:

“This work was supported by National Key R&D Program of China (2016YFA0502302 and 2017YFE0108200), by the Strategic Priority Research Program of the Chinese Academy of Sciences (XDB 20000000), by National Science Foundation of China (NSFC) under No. 91753119, 21472229 and 21778065, by the Fundamental Research Funds for the Central Universities, 2016YXMS261.”

