# Supplementary material for: Solution structure of extracellular loop of human β4 subunit of BK channel and its biological implication on ChTX sensitivity
Source: Sci Rep. 2018 Mar 15;8:4571. doi: 10.1038/s41598-018-23016-y (PMC5854672; doi:10.1038/s41598-018-23016-y)
Supplement: Supplementary file 1 — supplementary information [file 41598_2018_23016_MOESM1_ESM.docx]

**Solution structure of extracellular loop of human β4 subunit of BK channel and its biological implication on ChTX sensitivity**

Yanting Wang^1, 2*, ¥^, Wenxian Lan^1*^, Zhenzhen Yan^2^, Jing Gao^3^, Xinlian Liu^1^, Sheng Wang^2^, Xiying Guo^2^, Chunxi Wang^1^, Hu Zhou^3^, Jiuping Ding^2^, Chunyang Cao^1,4,5^

**Supplementary figures**

**Figure S1** The S-S bonds (**a**) between Cys68 and Cys119 and (**b**) between Cys54 and Cys148 were mapped through high-throughput mass spectrometry technique (also termed as pLink-SS).


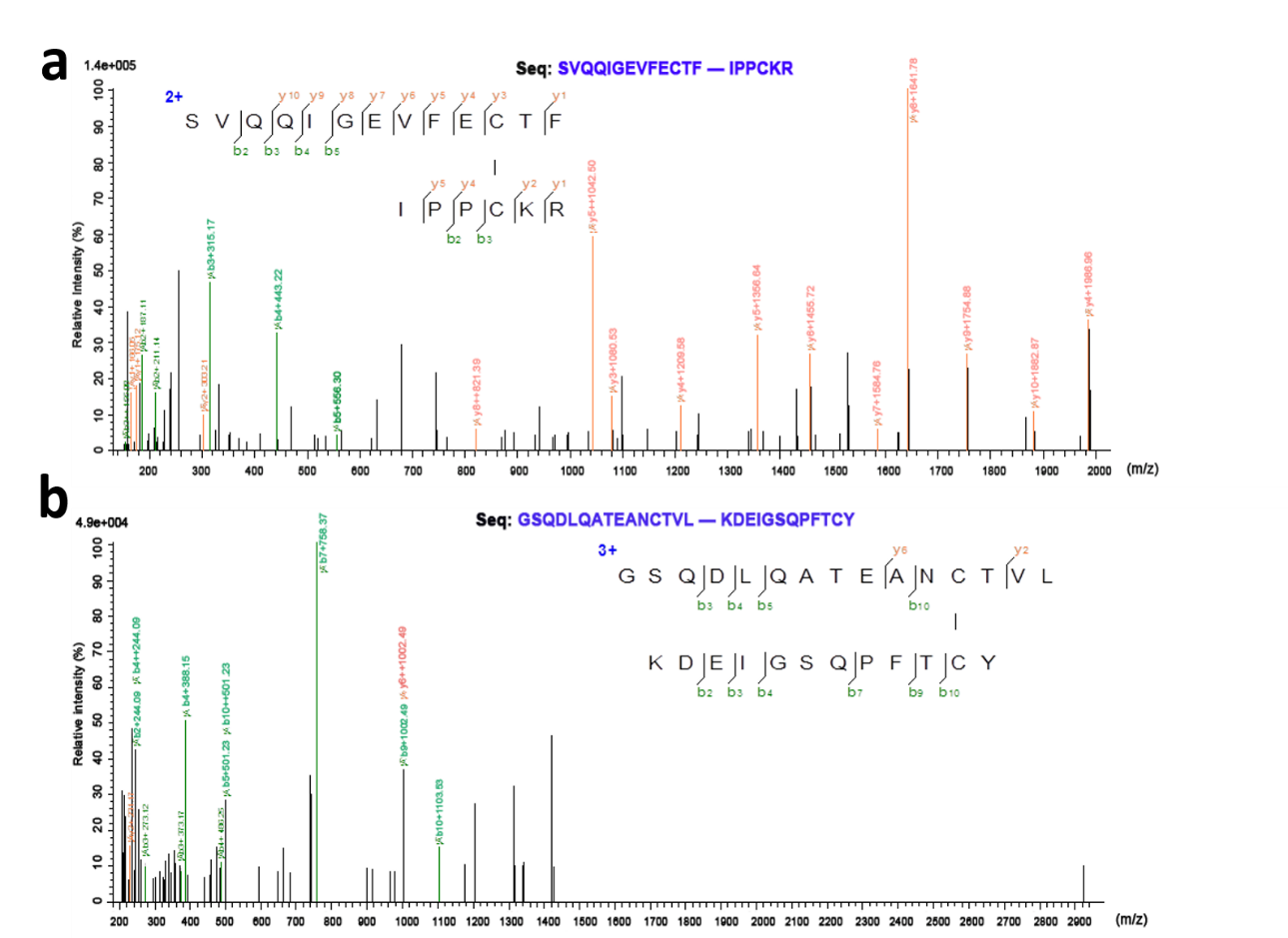


**Figure S2** The classic NOE patterns (displayed in arrows) observed between anti-parallel β-sheets in hβ4-loop. These NOEs confirmed all β-sheets conformations calculated by XPLOR program, and the constrains of hydrogen-bonds between these anti-parallel β-sheets were also setup based on these classic NOE patterns.


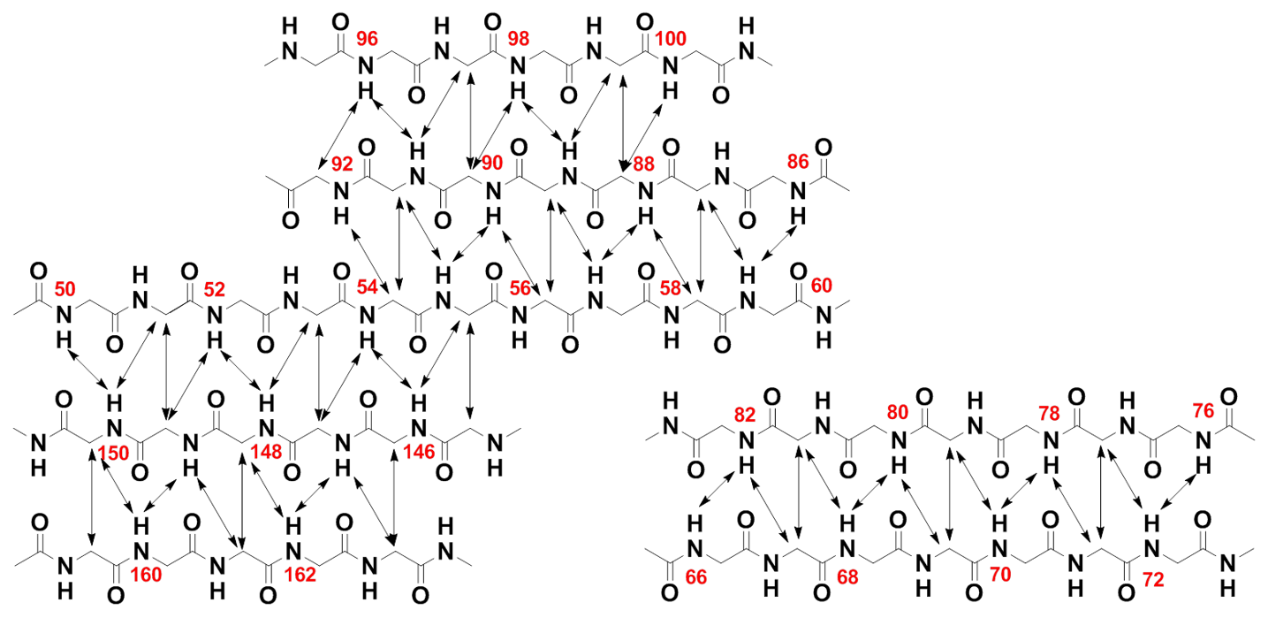


**Figure S3** The possible interaction sites in hβ4-loop with α-subunit detected by NMR titration experiments. (**a**) ^1^H-^15^N HSQC spectrum of free hβ4-loop; (**b-f)** Superposition of ^1^H-^15^N HSQC spectra of free hβ4-loop (in red) and of hβ4-loop bound with NH_2_ peptide of BKα subunit (in blue) (**b**); with S1-S2 linker of BKα subunit (in blue) (**c**); with S3-S4 linker of BKα subunit (in blue) (**d**); with S5-S6 linker 1 of BKα subunit (in blue) (**e**); and with S5-S6 linker 2 of BKα subunit (in blue) (**f**), respectively. (**b-f**) The cross-peaks with changes in chemical shifts or intensities were indicated with the assignments by arrows, compared to these in spectrum of free hβ4-loop in (**a**).


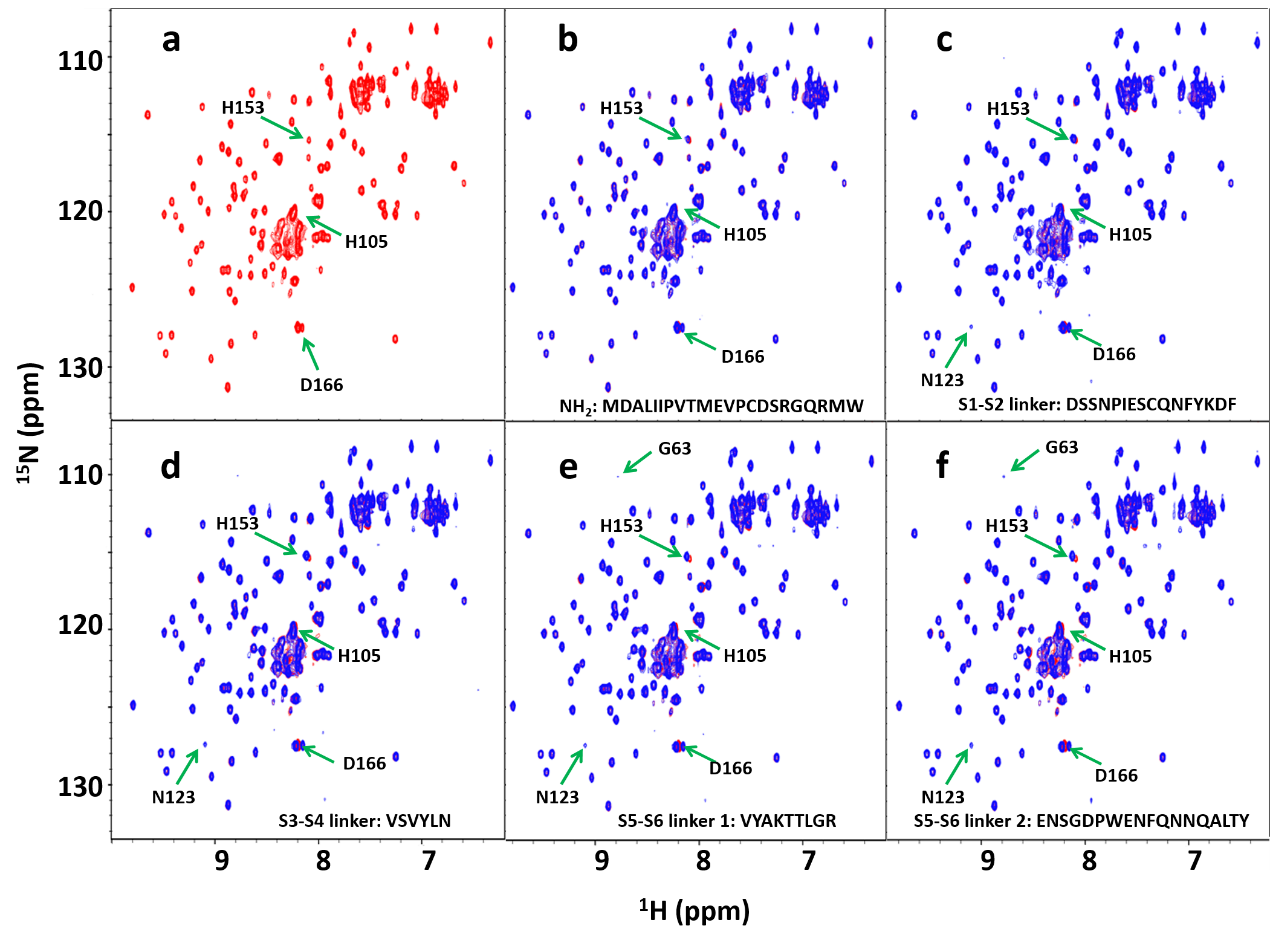


**Figure S4** The sequence alignment of BKα from *Aplysia californica* (5TJI isits cryo-EM structures in the absence of of Ca^2+^ ) and from human BKα. In hSlo1, the amino acids (1-20, 55-90 and 622-747) were deleted during construction of the open and close structural models.


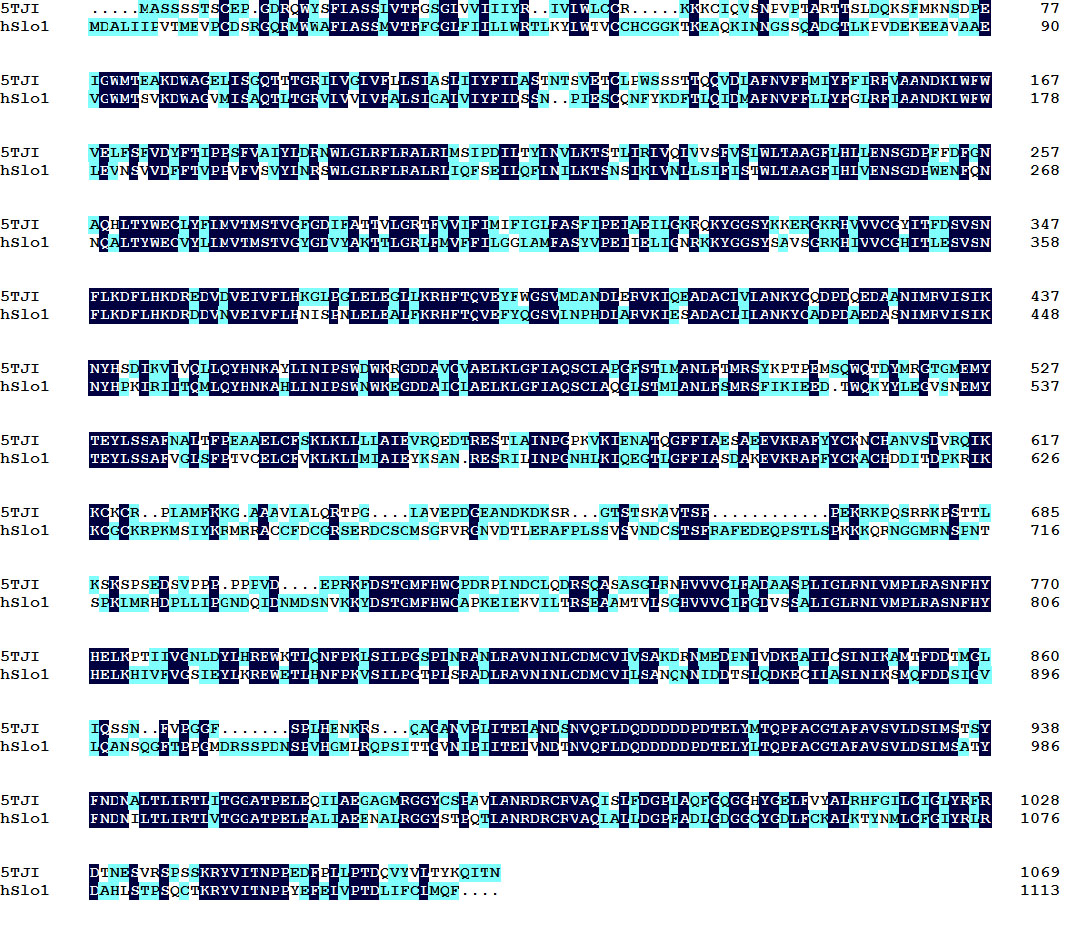


**Figure S5** Structural models of human BKα/β4 in complex with ChTX. (**a, d**) The structural models were constructed based on the cryo-EM structures of Slo1 from *Aplysia californica* in the absence of Ca^2+^ and in the presence of Ca^2+^, respectively [1, 2]. Ribbon representation of the assembled structural model of BKα/β4 channel in complex with ChTX, which composes four molecules of BKα subunit (displayed in pink, yellow, cyan and green, respectively), four molecules of β4 subunit (displayed in orange, grey, tints and red, respectively) and one molecule of ChTX (in blue). (**b, e**) To clearly view, ribbon representation of only four β4 subunits and one ChTX in the assembled structure models from (a) and (d), respectively. (**c, f**) The relative positions of ChTX (displayed in blue ribbon mode) and four-pairs of Asn123(β4)-Glu264(BKα) (displayed in stick mode) around ChTX in the assembled structure models from (a) and (d), respectively.


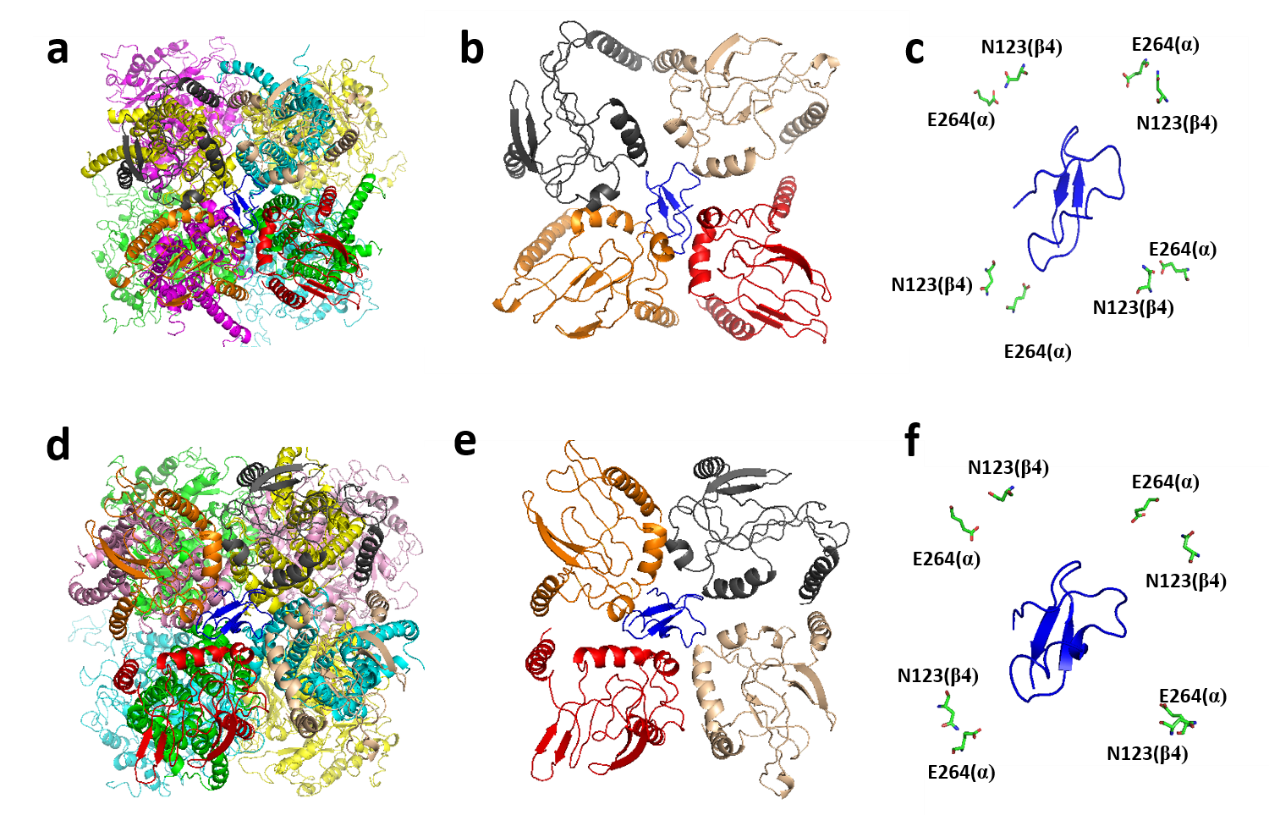


**Reference**

1. Hite, R.K., X. Tao, and R. MacKinnon, *Structural basis for gating the high-conductance Ca(2+)-activated K(+) channel.* Nature, 2017. **541**(7635): p. 52-57.

2. Tao, X., R.K. Hite, and R. MacKinnon, *Cryo-EM structure of the open high-conductance Ca2+-activated K+ channel.* Nature, 2017. **541**(7635): p. 46-51.

**Figure S6** Sequence comparison of BKα subunit derived from mouse (gi|309261855), monkey (gi|966962615), rabbit (gi|46396500) and human. The consensus Glu264 site was labeled with a red asterisk.

**
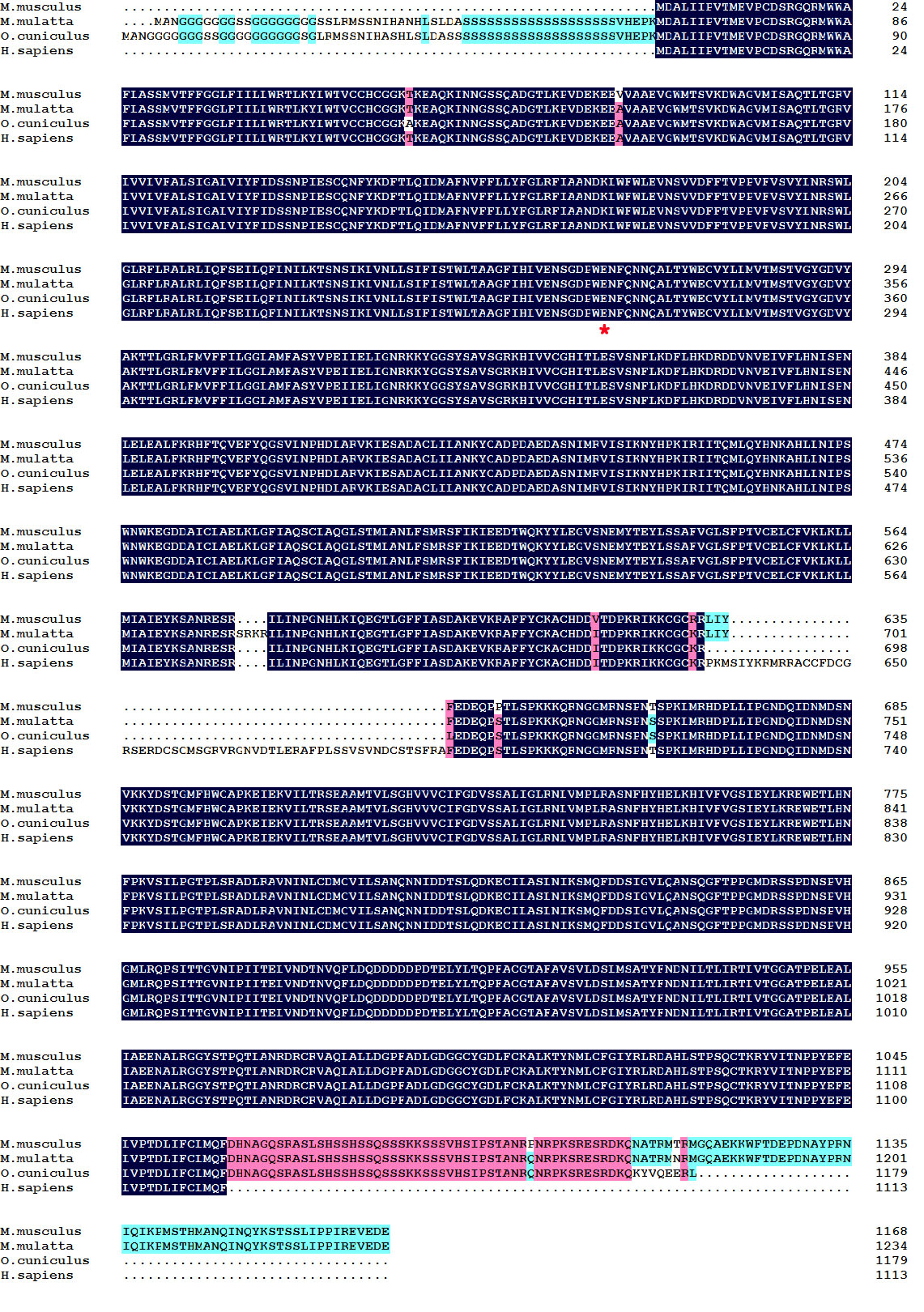
**

**Figure S7** Sequence comparison of transmembrane regions of β4 subunit derived from mouse (gi|10946834), rabbit (gi|291389559), human (gi|26051275) and monkey (gi|380811842).The consensus Asn123 site was labeled with a red asterisk.

**
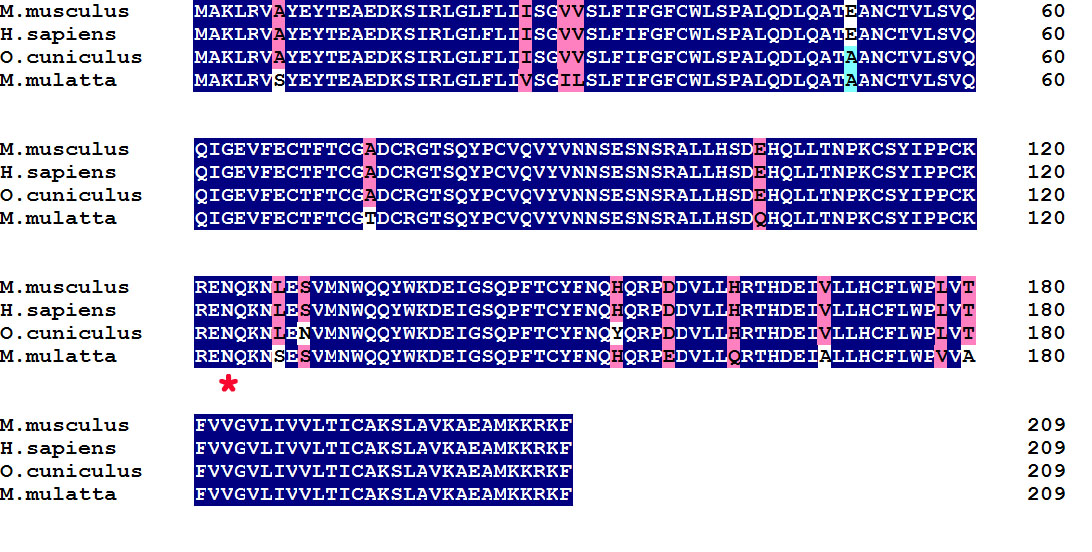
**
